# In-Depth Analysis of the Role of the Acinetobactin Cluster in the Virulence of *Acinetobacter baumannii*

**DOI:** 10.3389/fmicb.2021.752070

**Published:** 2021-10-05

**Authors:** Kelly Conde-Pérez, Juan C. Vázquez-Ucha, Laura Álvarez-Fraga, Lucía Ageitos, Soraya Rumbo-Feal, Marta Martínez-Guitián, Noelia Trigo-Tasende, Jaime Rodríguez, Germán Bou, Carlos Jiménez, Alejandro Beceiro, Margarita Poza

**Affiliations:** ^1^Servicio de Microbiología del Complejo Hospitalario Universitario de A Coruña (CHUAC), Instituto de Investigación Biomédica de A Coruña (INIBIC), A Coruña, Spain; ^2^Microbiome and Health, Faculty of Science, University of A Coruña, A Coruña, Spain; ^3^School of Chemistry and Molecular Biosciences, University of Queensland, Brisbane, QLD, Australia; ^4^Centro de Investigaciones Científicas Avanzadas (CICA) y Departamento de Química, Facultad de Ciencias, Agrupación Estratégica CICA-INIBIC, Universidad de A Coruña, A Coruña, Spain

**Keywords:** *Acinetobacter baumannii*, acinetobactin, fimsbactin, iron uptake, siderophore, virulence, mouse sepsis infection

## Abstract

*Acinetobacter baumannii* is a multidrug-resistant pathogen that represents a serious threat to global health. *A. baumannii* possesses a wide range of virulence factors that contribute to the bacterial pathogenicity. Among them, the siderophore acinetobactin is one of the most important, being essential for the development of the infection. In this study we performed an in-depth analysis of the acinetobactin cluster in the strain *A. baumannii* ATCC 17978. For this purpose, nineteen individual isogenic mutant strains were generated, and further phenotypical analysis were performed. Individual mutants lacking the biosynthetic genes *entA, basG*, *basC*, *basD*, and *basB* showed a significant loss in virulence, due to the disruption in the acinetobactin production. Similarly, the gene *bauA*, coding for the acinetobactin receptor, was also found to be crucial for the bacterial pathogenesis. In addition, the analysis of the Δ*basJ/*Δ*fbsB* double mutant strain demonstrated the high level of genetic redundancy between siderophores where the role of specific genes of the acinetobactin cluster can be fulfilled by their fimsbactin redundant genes. Overall, this study highlights the essential role of *entA*, *basG*, *basC*, *basD*, *basB* and *bauA* in the pathogenicity of *A. baumannii* and provides potential therapeutic targets for the design of new antivirulence agents against this microorganism.

## Introduction

*Acinetobacter baumannii* is one of the most common nosocomial pathogens responsible for a wide range of concerning diseases, such as pneumonia, bacteremia or secondary meningitis (Wong et al., 2017). The rise of healthcare-associated infections caused by multidrug resistant strains of *A. baumannii*, together with the scarce development of new antimicrobials in the last decades, represents an important health threat (De Oliveira et al., 2020). In fact, *A. baumannii*, is one of the Gram-negative ESKAPE pathogens identified by the World Health Organization (WHO) as critical priority for antibiotic discovery (World Health Organization [WHO], 2017).

Although the main characteristic of this pathogen is its ability to acquire new antimicrobial resistance, it shows several mechanisms involved in virulence, persistence and stress adaptation that enhance its pathogenicity (Harding et al., 2018). Within this context, many researchers have focused their efforts in developing alternatives to conventional antibiotics, such as antivirulence agents, that can work alone or together with antibiotics to overcome *A. baumannii* infections (Dickey et al., 2017).

**FIGURE 1:**
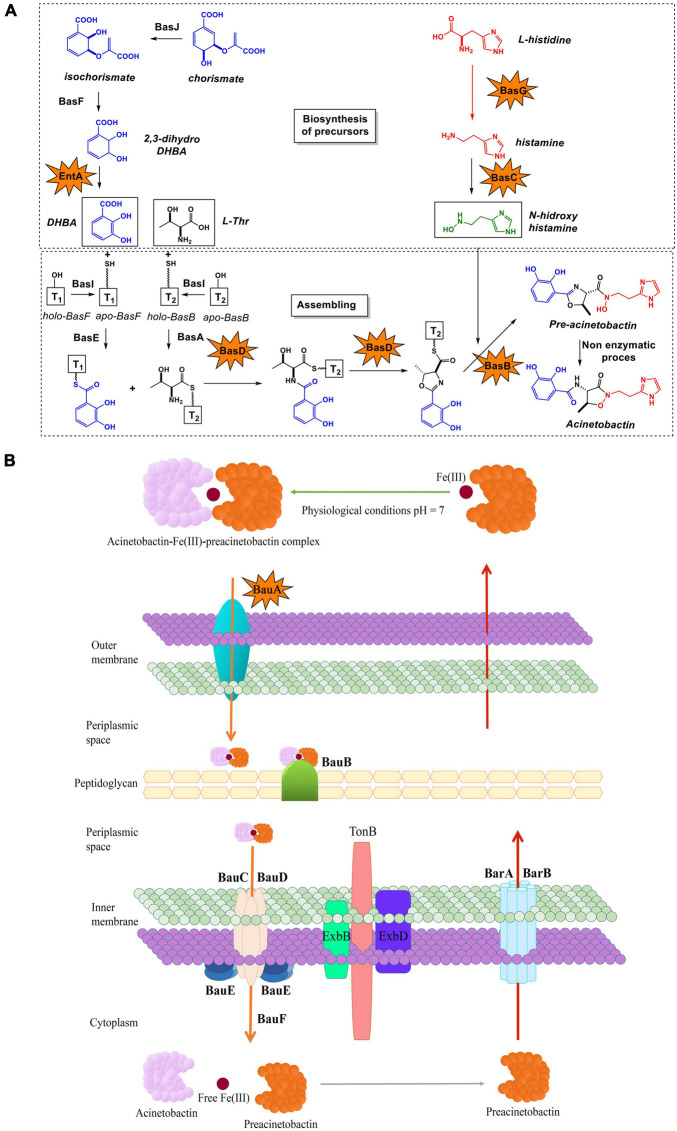
Proposed **(A)** biosynthetic pathway and **(B)** transport mechanism of acinetobactin in *A. baumannii*. The six proteins found to be essential for the development of the bacteremia infection are marked with stars.

Iron is an essential micronutrient for bacteria to infect and multiply in tissues and body fluids of the host, playing an important role in pathogenesis. The mechanisms of bacterial iron acquisition include: (i) expression of transporters involved in the uptake of ferrous iron, such as the Feo system; (ii) extraction of heme-iron from hemoproteins; (iii) capture of iron from transferrin and lactoferrin; or (iv) synthesis of siderophores (Sheldon et al., 2016). Siderophores are high-affinity iron-chelating molecules synthetized by microorganisms to scavenge extracellular ferric iron from the environment. Baumanoferrin, fimsbactin and preacinetobactin-acinetobactin (referred to as acinetobactin) are the most common siderophore systems detected in *A. baumannii* (Yamamoto et al., 1994; Proschak et al., 2013; Penwell et al., 2015). The most extensively studied is acinetobactin which is considered the major siderophore of *A. baumannii* and it is highly conserved among all *A. baumannii* strains (Antunes et al., 2011). All the genes required for the synthesis (*basA-J*), efflux (*barA/B*) and uptake (*bauA-F*) of acinetobactin are located in a 26.5-kb chromosomal region (Supplementary Figure 1A) (Mihara et al., 2004), with the exception of the *entA* homolog gene, found elsewhere in the chromosome (Penwell et al., 2012). Its biosynthesis follows the logic of a non-ribosomal peptide synthetase (NRPS) assembly system, where three precursors are bound in equimolar quantities into the preacinetobactin molecule: *N*-hydroxyhistamine, L-threonine and 2,3-dihydroxybenzoic acid (DHBA) (Figure 1A) (Yamamoto et al., 1994; Song and Kim, 2020). Once the preacinetobactin synthesis is completed, the siderophore is secreted to the extracellular space (Figure 1B) where two reactions can occur: (i) preacinetobactin stabilization by chelation of ferric iron or (ii) non-enzymatically and irreversibly isomerization to acinetobactin at pH > 7 (Shapiro and Wencewicz, 2016; Moynié et al., 2018). The fimsbactins A-F siderophores are present in a small fraction of the *A. baumannii* isolates (Antunes et al., 2011; Proschak et al., 2013). These siderophores, also derived from a NRPS assembly system, are structurally related to acinetobactin by the presence of catecholate, phenolate oxazoline, and hydroxamate metal-binding motifs (Proschak et al., 2013). Moreover, the cluster *fbsA-Q*, coding for the fimsbactins, consists of 18 genes with high functional similarity to those present in the acinetobactin cluster indicating redundancy between both siderophores pathways (Supplementary Figure 1B) (Dorsey et al., 2004; Mihara et al., 2004; Proschak et al., 2013). Although the expression of fimsbactins was shown to be enough to support the growth of *A. baumannii* in serum, these siderophores are not required for survival during bacteremia (Sheldon and Skaar, 2020).

Indeed, acinetobactin has been shown to be essential for the virulence of *A. baumannii* during greater wax moth (*Galleria mellonella)* and murine bacteremia and pneumonia infections (Gaddy et al., 2012; Penwell et al., 2012; Martínez-Guitián et al., 2020; Sheldon and Skaar, 2020). However, no studies have explored the contribution of the entire collection of acinetobactin genes to the infectious process.

Herein, we have performed an in-depth analysis of the role of the acinetobactin cluster in the virulence of *A. baumannii* which allowed us to identify potential targets for the design of new antimicrobials against this pathogen.

## Materials and Methods

### Bacterial Strains and Culture Conditions

All *A. baumannii* and *Escherichia coli* strains used in this study are listed in Table 1. Bacteria were grown routinely at 37°C in solid and liquid Luria-Bertani (LB) medium and stored at −80°C in LB broth containing 20% glycerol. When appropriate, media was supplemented with 50 μg/mL of kanamycin (Kan).

**TABLE 1:** Bacterial strains used in this work.

**Strain or plasmid**	**Relevant characteristics**	**Sources or references**
**Strains**		
***E. coli***		
TG1	Used for DNA recombinant methods	Lucigen
***A. baumannii***		
ATCC 17978	*A. baumannii* ATCC 17978 wild-type strain isolated from a fatal meningitis	American Type Culture Collection (ATCC)
Δ*basJ*	A1S_2372 gene deletion mutant from ATCC 17978	This study
Δ*basI*	A1S_2373 gene deletion mutant from ATCC 17978	This study
Δ*basH*	A1S_2374 gene deletion mutant from ATCC 17978	This study
Δ*barB*	A1S_2375 gene deletion mutant from ATCC 17978	This study
Δ*barA*	A1S_2376/77/78 gene deletion mutant from ATCC 17978	This study
Δ*basG*	A1S_2379 gene deletion mutant from ATCC 17978	This study
Δ*basF*	A1S_2380 gene deletion mutant from ATCC 17978	This study
Δ*basE*	A1S_2381 gene deletion mutant from ATCC 17978	This study
Δ*basD*	A1S_2382/83 gene deletion mutant from ATCC 17978	This study
Δ*basC*	A1S_2384 gene deletion mutant from ATCC 17978	This study
Δ*bauA*	A1S_2385 gene deletion mutant from ATCC 17978	This study
Δ*bauB*	A1S_2386 gene deletion mutant from ATCC 17978	This study
Δ*bauE*	A1S_2387 gene deletion mutant from ATCC 17978	This study
Δ*bauC*	A1S_2388 gene deletion mutant from ATCC 17978	This study
Δ*bauD*	A1S_2389 gene deletion mutant from ATCC 17978	This study
Δ*basB*	A1S_2390 gene deletion mutant from ATCC 17978	Martínez-Guitián et al., 2020
Δ*basA*	A1S_2391 gene deletion mutant from ATCC 17978	This study
Δ*bauF*	A1S_2392 gene deletion mutant from ATCC 17978	This study
Δ*entA*	A1S_2579 gene deletion mutant from ATCC 17978	This study
Δ*basJ/*Δ*fbsB*	A1S_2372 and A1S_2581 gene deletion double mutant from ATCC 17978	This study
Δ*basF/*Δ*fbsC*	A1S_2380 and A1S_2580 gene deletion double mutant from ATCC 17978	This study

### Construction of Isogenic Mutant Derivative Strains

All mutants were generated using the suicide vector pMo130 (Genbank: EU862243) as previously described (Álvarez-Fraga et al., 2016). Briefly, a PCR was performed to amplify both upstream and downstream regions flanking each gene of interest and cloned into the pMo130 vector. The plasmid constructions were electroporated into the wild-type strain *A. baumannii* ATCC 17978. Recombinant colonies representing the first crossover event were selected as previously described (Hamad et al., 2009). The second crossover event leading to gene knockout was confirmed by PCR followed by sequencing. All the primers used for the mutant construction are listed in the Supplementary Table 1.

### Growth Rate Analysis Under Normal and Iron-Limiting Conditions

Growth rates were assessed by measuring the optical density (OD) of the *A. baumannii* ATCC 17978 parental strain and the mutant derivative strains in Mueller Hinton II (MH) medium, in the presence (iron-limiting conditions) or absence (normal conditions) of 0.2 mM of the iron chelator 2,2′-bipyridyl (BIP), as previously described (Álvarez-Fraga et al., 2018). Growth was monitored at OD_600_ every 20 min until the late-log phase in 48-well plates using the Epoch 2 Microplate Spectrophotometer (BioTek Instruments, United States). The maximum specific growth rate (μ_max_) and the lag time (λ) parameters were calculated using the single Gompertz growth curve model (Tjørve and Tjørve, 2017). The maximum specific growth rate parameter represents the slope of the tangent at the inflection point. The lag time parameter represents x intercept of the μ_max_ tangent and shows the time (h) to enter exponential phase. Three independent biological replicates were carried out. Statistical analysis was performed using an unpaired, two tailed student’s *t*-test.

### Murine Sepsis Model

A murine sepsis model was used to evaluate the virulence of the *A. baumannii* ATCC 17978 parental strain and the isogenic mutant derivative strains as previously described (Martínez-Guitián et al., 2019). Briefly, groups of 10 female BALB/c mice were inoculated intraperitoneally with approximately 7.5 × 10^7^ colony forming units per mouse of exponentially grown cells and death was assessed during 168 h at 8-h intervals. The survival curves were plotted using the Kaplan-Meier method and analysed using the log-rank (Mantel-Cox) test. All experiments were carried out with the approval of and in accordance with the regulatory guidelines and standards established by the Animal Ethics Committee (Hospital Universitario A Coruña, Spain, project code P102).

### Chemical Analysis of the Siderophore Content of *Acinetobacter baumannii* Wild-Type and Mutant Strains

The siderophore content of *A. baumannii* wild-type and the mutant strains was analyzed by using our SPE-HLB/HPLC-HRMS methodology (Espada et al., 2011) adapted for the isolation of acinetobactin (Balado et al., 2015) and detecting the presence of iron(III) chelating compounds using the Chrome Azurol-S Liquid (CAS) assay. Briefly, bacteria were grown at 37°C in M9 minimal media supplemented with 0.2% casamino acids and 0.4% glucose until an OD_600_ = 1.0. Subsequently, the bacterial suspensions were pelleted, filtered and the resultant cell-free supernatants were freeze-dried to obtain 2.5 g of a residue. One gram of this material was dissolved in milli-Q water (1 mL), loaded in an OASIS HLB cartridge (6 g, 35 cm3, Waters), which was previously conditioned and equilibrated with 120 mL of acetonitrile (solvent B) and water (solvent A), each containing 0.1% TFA (v/v), and fractionated with 1:0, 9:1, 8:2, 7:3, and 0:1 of A:B (v/v) to give ABLH1-5 fractions, respectively. CAS-positive fractions were further analyzed by HPLC (Thermo Scientific) coupled to a PDA detector, monitoring the absorbance at λ = 254, 280 and 313 nm, and to a MSQ plus mass spectrometer in full positive ion mode. The analysis was carried out using a Discovery HS-F5 column (100 × 4.6 m, 5 μm), with a flow of 1 mL/min and the following gradient conditions: acetonitrile (solvent B) and water (solvent A), each containing 0.1% TFA (v/v), 40 min from 10 to 50% of B, 5 min from 50 to 100% of B, a 5 min of an isocratic step at 100% of B, 5 min from 100 to 10% of B and final 5 min of an isocratic step at 10% of B. HPLC/HRMS analysis of the ABLH3 fraction showed the presence of a chromatographic peak with a rt = 11.75 min, which presented a [M + H]^+^ adduct in its corresponding (+)-HR-ESIMS at *m/z* 347.1344 that agreed to that of acinetobactin (calcd. for C_16_H_19_N_4_O_5_, *m/z* 347.1350). In parallel, fimsbactins A and F were detected in the chromatographic peak with a rt = 19.4 min of ABLH5 by displaying the [M + H]^+^ adducts at *m/z* 575.1956 (calcd. for C_26_H_31_N_4_O_11_, 575.1989) and *m/z* 439.1803 (calcd. for C_19_H_27_N_4_O_8_, 439.1829) in the (+)-HR-ESIMS, respectively. Analogs of fimsbactins A and F, where the oxazoline ring is opened, were also identified in the chromatographic peak with a rt = 13.4 min by showing the [M + H]^+^ adducts at *m/z* 593.2063 (calcd. for C_26_H_33_N_4_O_12_, 575.2089) and *m/z* 457.1909 (calcd. for C_19_H_29_N_4_O_8_, 457.1229) in their (+)-HR-ESIMS. These analogs were formed from fimsbactins A and F due to the acidic conditions used for separation. Indeed, these compounds were not obtained when the same SPE-HLB/HPLC-MS methodology was used avoiding acidic conditions.

## Results

### Specific Genes of the Acinetobactin Cluster Are Relevant for the Growth of *Acinetobacter baumannii* ATCC 17978 Under Iron-Limiting Conditions

**FIGURE 2:**
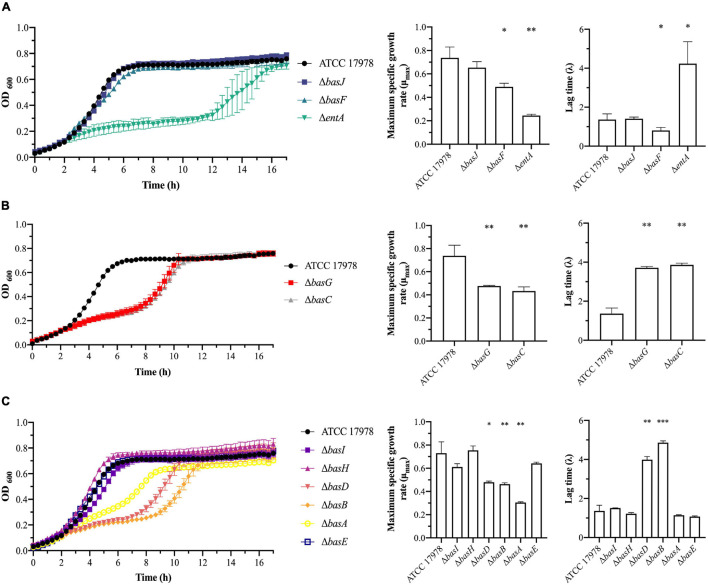
Growth curves and growth kinetics of *A. baumannii* ATCC 17978 and its isogenic mutant derivative lacking **(A)** genes involved in the synthesis of the DHBA precursor, **(B)** genes involved in the synthesis of the *N*-hydroxyhistamine precursor and **(C)** genes involved in the modification and assembly of the acinetobactin precursors into the final molecule. The growth curves were performed under iron-limiting conditions. Three independent biological replicates were performed. Unpaired student *t* test was used for the statistical analysis of the growth kinetics (^∗^*P* < 0.05; ^∗∗^*P* < 0.01; ^∗∗∗^*P* < 0.0001).

To determine the contribution of each gene of the acinetobactin cluster to the growth of *A. baumannii* ATCC 17978, a total of 19 individual isogenic mutant strains were generated (Figure 1, Table 1, Supplementary Figure 1, and Supplementary Table 2) and growth curves were performed in absence (normal conditions) and presence (iron-limiting conditions) of the iron (III) 2,2′- bipyridyl (BIP). The eleven mutant strains lacking the genes involved in the biosynthesis of acinetobactin were classified in three different groups: (i) genes involved in the synthesis of the DHBA precursor (*basJ, basF*, and *entA*), (ii) genes involved in the synthesis of the *N*-hydroxyhistamine precursor (*basG* and *basC*) and (iii) genes involved in the modification and assembly of the acinetobactin precursors into the final molecule (*basI, basH*, *basD, basB*, *basA*, and *basE*) (Figure 1A). The growth curves under iron-limiting conditions of the strains lacking the genes involved in the DHBA synthesis revealed that the Δ*entA* mutant was impaired for growth, with a lower maximum specific growth rate (μ_max_) (0.24, *P* = 0.0008) and a higher lag time (λ) (4.2, *P* = 0.0129) than the parental strain (μ_max_ = 0.74 and λ = 1.36). The deletion of *basF* also resulted in a slightly decrease of μ_max_ (0.49, *P* = 0.0119) and λ (0.81, *P* = 0.0423) (Figure 2A and Table 2). The mutant strains Δ*basG* and Δ*basC*, lacking genes involved in the synthesis of the *N*-hydroxyhistamine precursor, displayed a significant reduction in the μ_max_ (0.48, *P* = 0.0084 and 0.43, *P* = 0.0061; respectively) and an increase in the λ (3.7, *P* = 0.0002 and 3.9, *P* = 0.0001; respectively) when compared to the wild-type strain (Figure 2B and Table 2). Among the mutants belonging to the third group, Δ*basD*, Δ*basB*, and Δ*basA* grew poorly under iron-limitation conditions, showing significant lower μ_max_ (0.49, *P* = 0.01; 0.46, *P* = 0.0072 and 0.30, *P* = 0.0012; respectively). The Δ*basD* and Δ*basB* mutants also shown higher lag times (λ = 3.99, P = 0.0002 and λ = 4.86, *P* < 0.0001; respectively) compared to the wild-type strain (Figure 2C and Table 2). No significant differences in growth kinetics were observed in the other isogenic mutant strains [*P* (λ) = 0.16–0.82, *P* (μ_max_) = 0.09–0.74] compared to the wild-type strain ATCC 17978 (Figure 2). Under normal conditions, the eleven isogenic mutant strains showed similar growth abilities compared to the wild-type strain ATCC 17978 (Supplementary Figure 2A).

**TABLE 2:** Assays performed with the 21 isogenic mutant derivative strains.

**Mutant strains**	**Gene**	**Function**	**Fitness under iron-limiting conditions (μmax)**	**Fitness under iron-limiting conditions (λ)**	**Mice survival during sepsis**	**SPE-HLB/HPLC-MS**		
						**Acinetobactin**	**Fimsbactin A**	**Fimsbactin F**
**Mutant strains lacking genes involved in the acinetobactin biosynthesis**								
Δ*basJ*	A1S_2372	DHBA synthesis	−	−	−	d.	d.	d.
Δ*basF*	A1S_2380		+	+^a^	−	n.a	n.a	n.a
Δ*entA*	A1S_2579		++	+	++	n.a	n.a	n.a
Δ*basG*	A1S_2379	N-hydroxyhistamine synthesis	++	++	++	n.d.	d.	d.
Δ*basC*	A1S_2384		++	++	++	n.d.	d.	d.
Δ*basI*	A1S_2373	NRPS assembly system	−	−	−	n.a	n.a	n.a
Δ*basH*	A1S_2374		−	−	−	n.a	n.a	n.a
Δ*basD*	A1S_2382/83		+	++	++	n.d.	d.	d.
Δ*basB*	A1S_2390		++	+++	+++	n.d.	d.	d.
Δ*basA*	A1S_2391		++	−	−	n.a	n.a	n.a
Δ*basE*	A1S_2381		−	−	−	n.a	n.a	n.a
**Mutant strains lacking genes involved in acinetobactin transport**								
Δ*barA*	A1S_2376/77/78	Efflux	++	++	−	n.a	n.a	n.a
Δ*barB*	A1S_2375		++	+++	−	d.	d.	d.
Δ*bauA*	A1S_2385	Influx	−	−	++	d.	d.	d.
Δ*bauB*	A1S_2386		−	+	−	d.	d.	d.
Δ*bauC*	A1S_2388		+	++	−	n.a	n.a	n.a
Δ*bauD*	A1S_2389		+	−	−	n.a	n.a	n.a
Δ*bauE*	A1S_2387		+	−	−	n.a	n.a	n.a
Δ*bauF*	A1S_2392		−	−	−	n.a	n.a	n.a
**Double mutant strains**								
Δ*basJ/*Δ*fbsB*	A1S_2372/A1S_2581	DHBA synthesis	++	++	+++	n.d.	n.d	n.d
Δ*basF/*Δ*fbsC*	A1S_2380/A1S_2580		++	+++	+++	n.a	n.a	n.a

*Statistical significance of bacterial phenotype differences observed in the mutant strains compared with the parental ATCC 17978 strain are indicated as follows: +, *P* < 0.01;++, *P* < 0.05; +++, *P* < 0.0001 and −, no difference.*

*^a^Mutant decreased λ compared to the parental strain.*

*d. = detected.*

*n.d. = non-detected.*

*n.a = not analysed.*

**FIGURE 3:**
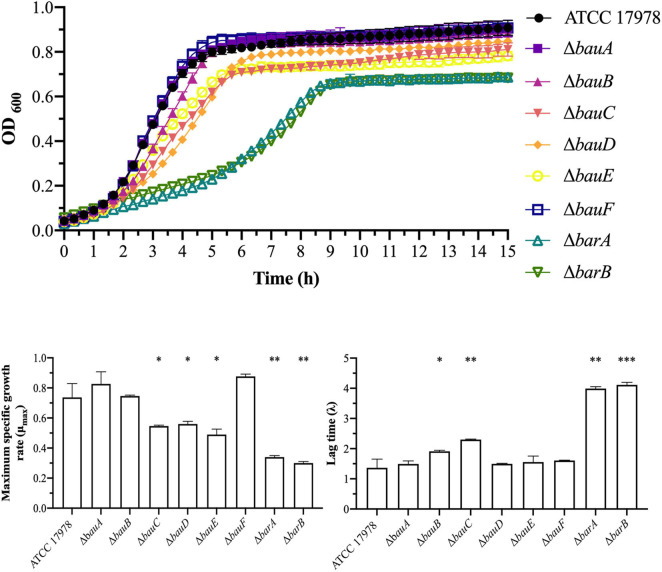
Growth curves and growth kinetics of *A. baumannii* ATCC 17978 and the isogenic mutant derivative strains lacking genes involved in the transport of acinetobactin. The growth curves were performed under iron-limiting conditions. Three independent biological replicates were performed. Unpaired student *t* test was used for the statistical analysis of the growth kinetics (^∗^*P* < 0.05; ^∗∗^*P* < 0.01; ^∗∗∗^*P* < 0.0001).

In parallel, we performed growth curves with the eight isogenic mutant derivative strains, lacking each of the influx and efflux related genes, under both normal and iron-limiting conditions. As it was observed in the mutant strains related to the acinetobactin biosynthesis, no significant differences were detected between the isogenic mutant strains and the wild-type strain under normal growth conditions (Supplementary Figure 2B). Nevertheless, under iron-limited conditions, the deletion of the genes *barA*, *barB* (efflux), and *bauC* (uptake) resulted in a significant growth inhibition shown as a reduction in the μ_max_ (0.34, *P* = 0.0018; 0.30, *P* = 0.0013 and 0.55, *P* = 0.0241; respectively) and an increase in the λ (3.99, *P* = 0.0001; 4.11, *P* < 0.0001 and 2.3, *P* < 0.0051; respectively) (Figure 3 and Table 2). We also observed a partial reduction of the growth of Δ*bauD* (μ_max_ = 0.56, *P* = 0.0320), Δ*bauE* (μ_max_ = 0.49, *P* = 0.0128) and Δ*bauB* (λ = 1.91, *P* < 0.0321) mutant strains growth. No significant differences in growth kinetics were observed in the other isogenic mutant strains [*P* (λ) = 0.22–0.51, *P* (μ_max_) = 0.06–0.86] (Figure 3).

### Six Genes of the Acinetobactin Cluster Are Essential for the Virulence *in vivo*

**FIGURE 4:**
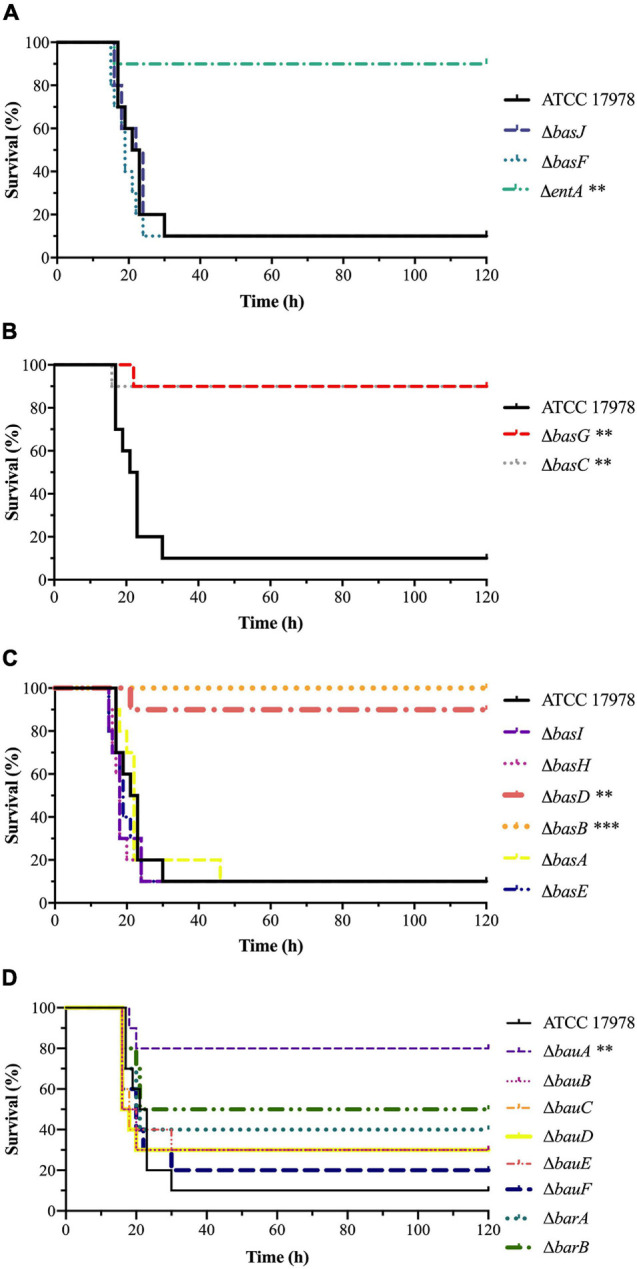
Sepsis infection in mice. Survival of BALB/c mice (*n* = 10 *per* group) after bacteremia infection with *A. baumannii* ATCC 17978 and the isogenic mutant derivative strains lacking **(A)** genes involved in the synthesis of the DHBA precursor, **(B)** genes involved in the synthesis of the *N*-hydroxyhistamine precursor, **(C)** genes involved in the modification and assembly of the acinetobactin precursors into the final molecule and **(D)** genes involved in the acinetobactin transport The log-rank (Mantel-Cox) test was used for statistical analysis (^∗∗^*P* < 0.01, ^∗∗∗^*P* < 0.0001).

It has been previously showed that acinetobactin plays an important role in the virulence of *A. baumannii* (Gaddy et al., 2012; Penwell et al., 2012; Martínez-Guitián et al., 2020; Sheldon and Skaar, 2020). However, it remains unclear which genes of the acinetobactin cluster are essential for the development of the infection. To elucidate this, a murine sepsis model was performed with the wild-type and the 19 mutant derivative strains. Among the mutant strains lacking genes involved in the biosynthesis of acinetobactin, the mice infected with Δ*entA* (90% survival, *P* = 0.0009), Δ*basG* (90% survival, *P* = 0.0004), Δ*basC* (90% survival, *P* = 0.0009),Δ*basD* (90% survival, *P* = 0.0004), and Δ*basB* (100% survival, *P* < 0.0001) mutant strains showed survival rates significantly higher compared with those of the mice infected with the parental strain (10% survival) (Figures 4A–C and Table 2). No significant differences in mice survival were observed in the other isogenic mutant strains (*P* = 0.3068–0.9716) (Figures 4A–C).

Among the mutant strains lacking genes involved in the transport of acinetobactin, only mice infected with the Δ*bauA* strain showed a significant increase in the survival rate (80% survival, *P* = 0.0039) compared with those mice infected with the parental strain (10% survival) (Figure 4D and Table 2). No significant differences in mice survival were observed in the other isogenic mutant strains (*P* = 0.1926–0.9484) (Figure 4D).

### Analysis of the Siderophore-Content of *Acinetobacter baumannii* Wild-Type and Mutant Strains

**FIGURE 5:**
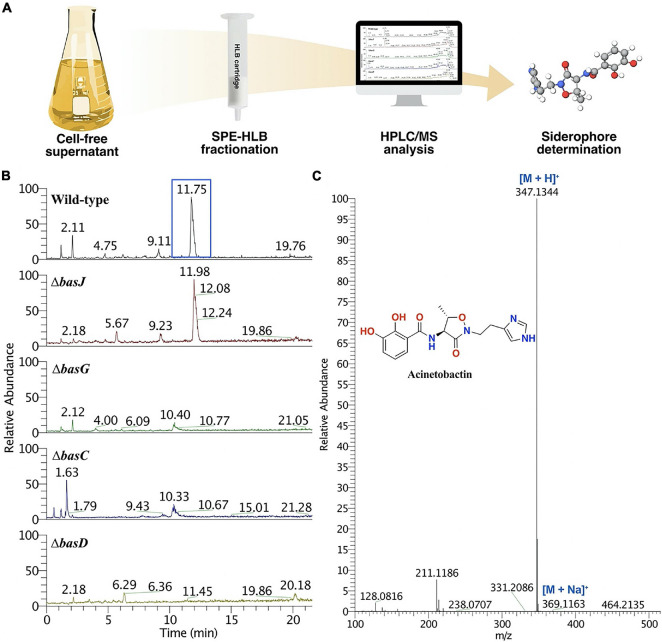
**(A)** Schematic representation of the siderophore-content isolation SPE-HLB/HPLC-MS methodology carried out in the cell-free supernatant of *A. baumannii* wild-type and mutant strains. **(B)** Comparison of the total ion current (TIC) chromatographic profiles of the ABLH3 fraction of *A. baumannii* wild type, in which acinetobactin was detected at rt = 11.75 min, to that of five mutant strains with depleted genes involved in the biosynthesis of acinetobactin. **(C)** High resolution mass spectrum of acinetobactin detected in the chromatographic peak at rt = 11.75 min of the ABLH3 fraction obtained from the *A. baumannii* wild-type strain.

To confirm whether the deletion of specific genes in *A. baumannii* ATCC 17978 caused a disruption in the biosynthesis or transport of acinetobactin, we analysed the presence of this siderophore in eight isogenic mutant strains (Table 2): Δ*basB*, Δ*basG*, Δ*basC*, Δ*basD*, and Δ*basJ*, related to the biosynthesis; Δ*bauA* and Δ*bauB*, related to the influx and Δ*barB*, related to the efflux of acinetobactin. We employed a bio-guided fractionation based on the SPE-HLB/HPLC-MS methodology described by Balado et al. (2015), using the colorimetric CAS liquid assay for the detection of iron(III)-chelating compounds (Supplementary Figure 3A). Thus, the cell-free supernatants of interest were freeze-dried and fractionated by solid-phase extraction (SPE) using hydrophilic-lipophilic balance (HLB) cartridges (Figure 5A). HPLC/HRMS analysis of the CAS-positive fractions obtained from the wild-type strain allowed us to detect acinetobactin and fimsbactins A and F (Figure 5A). Specifically, acinetobactin was localized in the chromatographic peak with rt = 11.75 min of the fraction ABLH3 eluted from the HLB cartridge with 8:2 of H_2_O:CH_3_CN (v/v), each containing 0.1% TFA (v/v), showing a [M + H]^+^ ion at *m/z* 347 in its corresponding MS (Figures 5B,C and Supplementary Figure 4). On the other hand, fimsbactin A and F were detected in the chromatographic peak with rt = 19.4 min of ABHL5 eluted with 100% of CH_3_CN, containing 0.1% TFA, displaying a [M + H]^+^ ion at *m/z* 575 and 439, respectively, in their MS (Supplementary Figures 5, 6). Due to the acidic conditions of the methodology, two analogs of fimsbactin A and F, having an opened oxazoline ring, were also found in the chromatographic peak with rt = 13.4 min of this fraction, displaying a [M + H]^+^ ion at *m/z* 593 and 457, respectively (Supplementary Figures 5, 7). This was confirmed after the analysis of the analogous fraction (ABLHWA5) obtained under non-acidic conditions (Supplementary Figures 3B, 8).

Finally, comparison of the HPLC chromatographic profiles of ABHL3 and ABHL5 fractions from the parental strain and the former selected mutant derivative strains, revealed that those mutants lacking genes involved in the biosynthesis of acinetobactin, except for Δ*basJ*, were not able to produce acinetobactin (Figure 5B, Table 2, and Supplementary Figure 9). However, the five mutant strains displayed the presence of fimsbactins A and F in their ABHL5 fractions (Table 2 and Supplementary Figure 10). In parallel, the mutant strains lacking genes involved in the influx and efflux of acinetobactin did not show any difference in comparison to the parental strain in none of the fractions (Supplementary Figures 11–14).

### Specific Genes of the Fimsbactin Cluster Could Contribute to the Acinetobactin Biosynthesis

Our results showed that the genes *basG* and *basC*, involved in the biosynthesis of the *N*-hydroxyhistamine precursor, and the genes *basD* and *basB*, involved in the assembly of the precursors, are essential for the biosynthesis of acinetobactin and therefore, they are crucial for the virulence of *A. baumannii in vivo*. However, the deletion of *basJ* gene which is involved in the biosynthesis of the DHBA precursor did not have any effect in the biosynthesis of acinetobactin and in the virulence of the bacterium. Taking into account that DHBA is also a fimsbactin precursor, our data suggest that in the absence of specific genes of the acinetobactin cluster, *A. baumannii* ATCC 17978 is able to successfully synthetize acinetobactin using redundant genes from the fimsbactin cluster.

To explore this hypothesis, we performed an in-depth analysis of both clusters using BLAST (see Supplementary Figure 1 for cluster organization and Supplementary Table 2 for gene description). This analysis showed that all the genes (except for *entA*) belonging to the acinetobactin cluster involved in the DHBA biosynthesis and the NRPS assembly have a potential redundant gene in the fimsbactin cluster (Supplementary Table 3). To further investigate this genetic redundancy, *basJ* and *basF* genes were selected and two double mutant strains were generated lacking both redundant genes of the acinetobactin and the fimsbactin clusters (Δ*basJ/*Δ*fbsB* and Δ*basF/*Δ*fbsC)* (Table 1).

**FIGURE 6:**
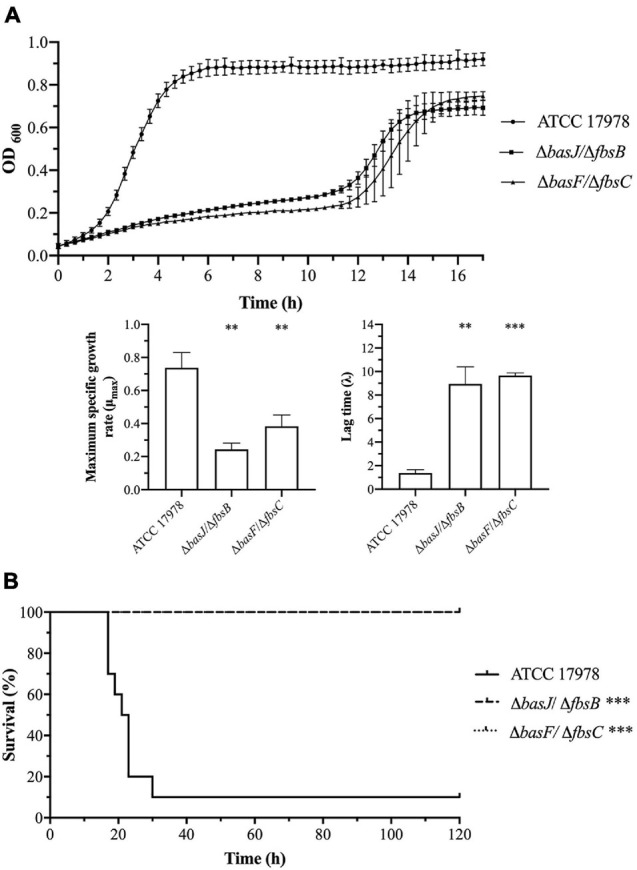
**(A)** Growth curves and growth kinetics of *A. baumannii* ATCC 17978 and the Δ*basJ/*Δ*fbsB* and Δ*basF/*Δ*fbsC* double mutant strains. The growth curves were performed under iron-limiting conditions. **(B)** Sepsis infection in mice. Survival of BALB/c mice (*n* = 10 *per* group) after bacteremia infection with *A. baumannii* ATCC 17978 and the Δ*basJ/*Δ*fbsB* and Δ*basF/*Δ*fbsC* double mutant strains. The log-rank (Mantel-Cox) test was used for statistical analysis (^∗∗^*P* < 0.01, ^∗∗∗^*P* < 0.0001).

Growth curves under iron-limiting conditions showed a significant decrease in the growth abilities of both Δ*basJ/*Δ*fbsB* (μ_max_ = 0.24, *P* = 0.001 and λ = 8.94, *P* = 0.0009) and Δ*basF/*Δ*fbsC* (μ_max_ = 0.38, *P* = 0.006 and λ = 9.65, *P* < 0.0001) mutant strains compared to the parental strain ATCC 17978 (μ_max_ = 0.74 and λ = 1.36) (Figure 6A and Table 2). However, no significant differences were detected between the isogenic double mutant strains and the wild-type strain under normal growth conditions (Supplementary Figure 2C).

Furthermore, a murine sepsis model was performed with the ATCC 17978 parental strain and the two double isogenic mutant strains. Mice infected with Δ*basJ/*Δ*fbsB* and Δ*basF/*Δ*fbsC* strains displayed a significantly increase of the survival rate (100% survival, *P* < 0.0001) in relation to those infected with the ATCC 17978 parental strain (10% survival) (Figure 6B and Table 2).

Finally, the siderophore-content of the double mutant Δ*basJ/*Δ*fbsB* was studied using our SPE-HLB/HPLC/MS methodology, revealing complete inhibition of acinetobactin and fimsbactins production by lack of detection of these siderophores in the HPLC/MS analysis (Table 2 and Supplementary Figures 13, 14).

## Discussion

In the last decades, the emergence of *A. baumannii* multidrug-resistant strains has become a worldwide concerning problem derived from the scarcity of effective therapeutic options against this bacterium. Hence, the World Health Organization (WHO) included *A. baumannii* as a critical priority pathogen claiming an urgent need of efficient alternatives to the known antibiotics (World Health Organization [WHO], 2017). Within this context, the search and identification of new therapeutic targets in *A. baumannii* have become a priority.

The pathogenesis success of *A. baumannii* is partially linked to the synthesis of active siderophores that supply the iron needed for its essential role in crucial metabolic events. Among all the siderophore systems identified, acinetobactin is considered the major siderophore of this bacterium (Yamamoto et al., 1994). Since then, several studies have focused on unraveling its regulation, chelating mechanisms and its role in the virulence of the bacterium (Mihara et al., 2004; Gaddy et al., 2012; Shapiro and Wencewicz, 2016; Sheldon and Skaar, 2020). After confirming that acinetobactin-related metabolism is a crucial virulence factor and that it is highly conserved among *A. baumannii* strains, the iron(III) uptake system mediated by acinetobactin has been proposed as a potential therapeutic target to combat this multidrug-resistant pathogen (Antunes et al., 2011; Gaddy et al., 2012; Sheldon and Skaar, 2020). However, the contribution of each individual gene involved in the acinetobactin metabolism in the infectious process is still unclear. Thus, we have performed an in-depth analysis of the acinetobactin gene cluster by conducting different phenotypical assays with the well-known strain *A. baumannii* ATCC 17978 and 19 isogenic mutant derivative strains lacking genes involved in the biosynthesis and transport of acinetobactin.

Three different siderophore-mediated iron uptake systems (acinetobactin, baumanoferrin and fimsbactin) were identified from the reference strain *A. baumannii* ATCC 17978, which corresponding gene clusters were found upregulated under *in vitro* iron-limiting conditions and *in vivo* infection (Eijkelkamp et al., 2011; Murray et al., 2017; Martínez-Guitián et al., 2020). Acinetobactin and fimsbactins are synthesized through non-ribosomal peptide synthetase (NRPS) assembly systems, sharing the DHBA and L-threonine precursors (Proschak et al., 2013; Song and Kim, 2020). This could explain the high level of genetic redundancy between both clusters where most of the genes involved in the biosynthesis of the acinetobactin have a potential redundant gene in the fimsbactin cluster. Hence, although fimsbactins are only present in a small percentage of *A. baumannii* strains, fimsbactin genes could complement the inactivation of some acinetobactin genes, suggesting a redundancy in both pathways. Within this context, *A. baumannii* ATCC 17978 confers the perfect background for the present study.

Bioinformatic analysis of the acinetobactin gene cluster showed that *basG, basC* and *entA* genes do not have a potential redundant gene. The lack of redundant *basG* and *basC* genes in the fimsbactin cluster is easily explained since these genes are involved in the biosynthesis of the precursor of acinetobactin *N*-hydroxyhistamine (Shapiro and Wencewicz, 2016) and this moiety is not present in the fimsbactins. On the other hand, the *entA* gene is always located outside of the acinetobactin cluster, varying its location between strains. In *A. baumannii* ATCC 17978, acinetobactin and fimsbactins share the gene *entA*, which is located in the fimsbactin cluster (Penwell et al., 2012). The individual deletion of these three genes resulted in a drastic reduction of the virulence when compared to the wild-type strain. Our results agreed with a recent study published by Sheldon and Skaar where they demonstrated that the deletion of gene *basG* impairs growth on human serum, transferrin or lactoferrin as sole iron sources, and severely attenuates survival of *A. baumannii* ATCC 17978 in a murine bacteremia model (Sheldon and Skaar, 2020).

Although Dorsey et al. predicted that *basC* gene had an essential function in the biosynthesis of the acinetobactin on the basis of its involvement in the synthesis of *N*-hydroxyhistamine (Dorsey et al., 2004), this hypothesis was never investigated until now. Siderophore-content analysis of the cell free supernatants of both Δ*basG* and Δ*basC* using our SPE-HLB/HPLC-MS methodology showed that the deletion of these genes resulted in the complete inhibition of acinetobactin production. The low virulent phenotype of these two mutants could be related to the lack of the siderophore. As the previous case, it would be expected that the deletion of the *entA* gene will inhibit the biosynthesis of both acinetobactin and fimsbactin siderophores.

Deletion of *basB* and *basD* also led to a significant decrease in virulence characterized by an impaired fitness under iron-limiting conditions and increased mice survival. We have previously reported that *basB* is an essential gene for the virulence of *A. baumannii* during pneumonia in mice and for bacteria growth under iron-limiting conditions (Martínez-Guitián et al., 2020). In addition, Gaddy et al. demonstrated that *basD* gene is essential for the biosynthesis of acinetobactin and for the bacterial growth under iron-depleted conditions in *A. baumannii* ATCC 19606 (Gaddy et al., 2012). Both genes code for proteins involved in the last steps of the biosynthetic pathway of acinetobactin, where DHBA and L-Threonine precursors are linked (BasD) and the resulting intermediate is bonded to *N*-hydroxyhistamine (BasB) to give preacinetobactin (Hasan et al., 2015; Shapiro and Wencewicz, 2016; Song and Kim, 2020). Both Δ*basB* and Δ*basD* mutants were unable to synthetize acinetobactin.

A closer analysis of the *basJ* gene, showed that its deletion did not have any effect in the biosynthesis of acinetobactin. However, the deletion of both acinetobactin (*basJ*) and fimsbactin (*fbsB*) redundant genes resulted in the loss of acinetobactin and fimsbactins production. This fact demonstrates that in absence of the *basJ* gene, *A. baumannii* ATCC 17978 can use *fbsB* to synthesize acinetobactin. This is a clear example of molecular redundancy whereby two genes have the same function or when an alternative pathway fulfills the mission role of an inactivated gene. Pathogens used it to adapt to a continuous changing environment, avoiding the antimicrobial defenses of their hosts (Ghosh and O’Connor, 2017). Based on our results, we predict that the redundancy of Δ*basF*,Δ*basI*,Δ*basH*,Δ*basA*, and Δ*basE* strains possibly lead to the unchanged ability to synthesize acinetobactin.

Among the genes involved in the transport of acinetobactin, only the gen *bauA*, coding for the outer-membrane receptor, was found to be essential for the virulence during a murine sepsis model. Previous studies have shown that the gene *bauA* is essential for the virulence of *A. baumannii* ATCC 19606 since its deletion led to a decrease in the ability of the bacteria to infect, divide inside body fluids of mice and in fitness under iron-limiting conditions (Gaddy et al., 2012). In fact, BauA was proposed as a good vaccine candidate since mice injected with recombinant BauA were able to produce antibodies against this protein. In addition, passive immunization using serum anti-BauA protected mice from infection (Esmaeilkhani et al., 2016). Our data slightly differ with this study since we have not observed any reduction in the fitness of *A. baumannii* ATCC 17978 when *bauA* was deleted. This discrepancy could be explained by the higher susceptibility of the strain ATCC 19606 to chelate iron (III) compared to the ATCC 17978 strain, possibly due to the lack of fimsbactins and baumanoferrin production in the ATCC 19606 strain (Antunes et al., 2011; Proschak et al., 2013; Penwell et al., 2015; Ramirez et al., 2019).

Both BarA and BarB proteins belong to the acinetobactin secretion system (efflux) of the ABC superfamily. Mutant strains lacking the genes involved in the synthesis of these proteins, Δ*barA* and Δ*barB*, showed a significant decrease in fitness under iron-limiting conditions. Notwithstanding, no statistical differences between the percentage of survival of the mice infected with these mutant strains and the mice infected with the parental strain were observed. SPE-HLB/HPLC-MS analysis of Δ*barB* mutant strain cultures showed the presence of acinetobactin in its cell-free supernatant, which indicates that this single mutant strain did not prevent the efflux of acinetobactin outside the bacteria. A previous study carried out by Penwell et al. in *A. baumannii* ATCC 19606, showed a growth defect under iron-limiting conditions and a 60% decrease of acinetobactin effluxed in the Δ*barA*/Δ*barB* cell-free supernatant compared with the parental strain (Penwel, 2013). The reduction in the efflux of acinetobactin matches with the partial loss of virulence in the Δ*barA* and Δ*barB* mutant strains. It is known that *A. baumannii* possesses a wide variety of transport mechanisms. It is possible that under stress conditions, the bacteria could use non-specific transporter systems to secrete and uptake acinetobactin and do not lose the iron-battle against the host (Iacono et al., 2008; Coyne et al., 2010, 2011; Fernando and Kumar, 2012).

Several researchers have focused their efforts on the development of new inhibitors based on acinetobactin metabolism. Inhibitors of BasE, an enzyme involved in biosynthesis of the acinetobactin, have shown a powerful inhibitory activity (Neres et al., 2013). Analogous of acinetobactin have also shown bacteriostatic activity as they were able to block the transport of the iron-acinetobactin complex inside the bacteria (Bohac et al., 2017; Shapiro and Wencewicz, 2017). In the last years, siderophore conjugates using the “Trojan Horse” antibiotic drug delivery strategy has become more popular for combating this microorganism. In fact, cefiderocol (fetcroja) was the first cathecol-substituted siderophore cephalosporin approved by the FDA and EMA (Ji et al., 2012; Wencewicz and Miller, 2013; Ghosh et al., 2017; Parsels et al., 2021).

In summary, we performed an in-depth analysis of the role of each individual gene of the acinetobactin metabolism in the virulence of *A. baumannii* ATCC 17978, allowing us to identify six potential targets for the design of new antimicrobials against this microorganism: five of them involved in its biosynthesis (*entA*, *basG, basC*, *basD*, and *basB*) and one related to its transport (*bauA*). Due to the similar function and potentially similar structure of the enzymes involved in the biosynthesis of acinetobactin and fimsbactin, inhibitors against the remaining biosynthetic steps could also have the potential to be effective by inactivating both redundant proteins.

## Data Availability Statement

The original contributions presented in the study are included in the article/Supplementary Material, further inquiries can be directed to the corresponding author.

## Ethics Statement

The animal study was reviewed and approved by Hospital Universitario A Coruña, Spain, project code P102.

## Author Contributions

KC-P, SR-F, NT-T, and LÁ-F performed mutant construction. MM-G, JV-U and KC-P performed phenotypic experiments and animal models. LA performed the analysis of the siderophore-content. AB, MP, CJ, and LÁ-F designed and supervised the experiments and wrote the manuscript. GB and JR revised the manuscript. All authors read and approved the final manuscript.

## Conflict of Interest

The authors declare that the research was conducted in the absence of any commercial or financial relationships that could be construed as a potential conflict of interest.

## Publisher’s Note

All claims expressed in this article are solely those of the authors and do not necessarily represent those of their affiliated organizations, or those of the publisher, the editors and the reviewers. Any product that may be evaluated in this article, or claim that may be made by its manufacturer, is not guaranteed or endorsed by the publisher.
