# Supplementary material for: In-Depth Analysis of the Role of the Acinetobactin Cluster in the Virulence of Acinetobacter baumannii
Source: Front Microbiol. 2021 Oct 5;12:752070. doi: 10.3389/fmicb.2021.752070 (PMC8524058; doi:10.3389/fmicb.2021.752070)
Supplement: Supplementary file 1 [file Table_1.DOCX]

**Supplementary Table 1.** Primers used in the present work.

| **Primer** | **Sequence** | **Use in the present study** |
| --- | --- | --- |
| A1S_2372UPFw | gggctgcagttacatggacattagatagct | Construction of the Δ*basJ* mutant strain |
| A1S_2372UPRv | ggggatatccagaagctaaaatgcaaacc |  |
| A1S_2372DOWNFw | ggggatatctctgtatatcacctctcaca |  |
| A1S_2372DOWNRv | gggggatccgcatggcaacaacacacag |  |
| A1S_2372intUP | gtattcacggtacaccaaa | Confirmation of the Δ*basJ* mutant strain |
| A1S_2372intDOWN | ctagatggcacgtttcttta |  |
| A1S_2373UPFw | gggctgcaggtttaaggcatcgggag | Construction of the Δ*basI* mutant strain |
| A1S_2373UPRv | gggatatcattacaatttcttgcattga |  |
| A1S_2373DOWNFw | ggggatatcagcatggactttattcaata |  |
| A1S_2373DOWNRv | gggggatccatgcgccgattttaatact |  |
| A1S_2373intUP | ggtatttgcagcaggtg | Confirmation of the Δ*basI* mutant strain |
| A1S_2373intDOWN | gactgaggttccgatc |  |
| A1S_2374UPFw | gggctgcagaggtgtatttaacgttttaa | Construction of the Δ*basH* mutant strain |
| A1S_2374UPRv | ggggagctcagtccatgctatgaatac |  |
| A1S_2374DOWNFw | ggggagctcattatattgatgtttattcac |  |
| A1S_2374DOWNRv | gggggatccgagacattagtacggatc |  |
| A1S_2374intUP | gcgttgtaatgcatatttt | Confirmation of the Δ*basH* mutant strain |
| A1S_2374intDOWN | tgccttggatgaagaga |  |
| A1S_2375UPFw | gggctgcagctaattgacgaatgaagcgt | Construction of the Δ*barB* mutant strain |
| A1S_2375UPRv | ggggatatctaaaaaaatagagcagaaatc |  |
| A1S_2375DOWNFw | ggggatatcattcataaagatttccctgag |  |
| A1S_2375DOWNRv | gggggatccatgtatcaaaacgggatgg |  |
| A1S_2375intUP | ccaatagatcaggatcatca | Confirmation of the Δ*barB* mutant strain |
| A1S_2375intDOWN | gtgaaaaacagcgtatagga |  |
| A1S_2376/77/78UPFw | attgatatgcaaccctgcag | Construction of the Δ*barA* mutant strain |
| A1S_2376/77/78UpRv | cgtgcgtttttatcctcagc |  |
| A1S_2376/77/78DOWNFw | gcttatctctctttatattca |  |
| A1S_2376/77/78DOWNRv | cgccaaattattgcagatga |  |
| A1S_2376/77/78intUP | agtcagttgggcctttaag | Confirmation of the Δ*barA* mutant strain |
| A1S_2376/77/78intDOWN | aacgtacagttggcttttag |  |
| A1S_2379UPFw | gggctgcagcgattatcacgaatgcc | Construction of the Δ*basG* mutant strain |
| A1S_2379UPRv | ggggagctctttccgtgtccttctga |  |
| A1S_2379DOWNFw | ggggagctctttttgctccgttaatatg |  |
| A1S_2379DOWNRv | gggggatcctattgagcagcatatggc |  |
| A1S_2379intUP | taaccaccctatcaaaata | Confirmation of the Δ*basG* mutant strain |
| A1S_2379intDOWN | gcagttttcttcggaac |  |
| A1S_2380UPFw | ggggcggccgcatttttagacaatgttgaatt | Construction of the Δ*basF* mutant strain |
| A1S_2380UPRv | ggggagctcagggttgccatcataag |  |
| A1S_2380DOWNFw | ggggagctcatgctgctcaatatttatta |  |
| A1S_2380DOWNRv | gggggatccctacgactatagtgtgc |  |
| A1S_2380intUP | aatagagaatttgaaaaaacg | Confirmation of the Δ*basF* mutant strain |
| A1S_2380intDOWN | agttatacacgcagctttg |  |
| A1S_2381UPFw | gggctgcagggagtcgctatcaaagatat | Construction of the Δ*basE* mutant strain |
| A1S_2381UPRv | ggggatatcaaagtttgccactgaccg |  |
| A1S_2381DOWNFw | ggggatatctcaactgttttttcatgacatt |  |
| A1S_2381DOWNRv | gggggatccaccaagcgtataggcaaata |  |
| A1S_2381intUP | taaggctgtgctgtataga | Confirmation of the Δ*basE* mutant strain |
| A1S_2381intDOWN | aattgaccgtcaaattctgc |  |
| A1S_2382/83UPFw | ggggcggccgcgtattcgagtttaaaccgca | Construction of the Δ*basD* mutant strain |
| A1S_2382/83UPRv | ggggtcgacgtcgttgaagtgctaaatgt |  |
| A1S_2382/83DOWNFw | ggggtcgacattcgtattttgaactttcatc |  |
| A1S_2382/83DOWNRv | gggggatccacccgattcaagttctttaa |  |
| A1S_2382/83intUP | ccagcgaacaaactcaatc | Confirmation of the Δ*basD* mutant strain |
| A1S_2382/83intDOWN | caatagcaacccttcatgc |  |
| A1S_2384UPFw | gggctgcagacagttgtctaaaattggcg | Construction of the Δ*basC* mutant strain |
| A1S_2384UPRv | ggggatatcgtttatactttatctgtttcc |  |
| A1S_2384DOWNFw | ggggatatcaaaaacgcttaataaaaaaaac |  |
| A1S_2384DOWNRv | gggggatccgggagatcgtgtaactaata |  |
| A1S_2384intUP | gtggacaactcaactcattt | Confirmation of the Δ*basC* mutant strain |
| A1S_2384intDOWN | actattcgcgggaatattga |  |
| A1S_2385UPFw | ggggcggccgcgattgtcgatttggagaaaa | Construction of the Δ*bauA* mutant strain |
| A1S_2385UPRv | ggggatatctattatctgccacaattgat |  |
| A1S_2385DOWNFw | ggggatatcgacctttaatgttttgattc |  |
| A1S_2385DOWNRv | gggctgcagtgaactggaagaaaaagtat |  |
| A1S_2385intUP | tgatctataaagggattagc | Confirmation of the Δ*bauA* mutant strain |
| A1S_2385intDOWN | tttctgaacttgatgaacag |  |
| A1S_2386UPFw | ggggcggccgcatcatttaagtcatactcgc | Construction of the Δ*bauB* mutant strain |
| A1S_2386UPRv | ggggatatcgtgaaaaagggctatcaata |  |
| A1S_2386DOWNFw | ggggatatcgttcatatgtattcaagctc |  |
| A1S_2386DOWNRv | gggctgcagcgctatgtcagtttctattt |  |
| A1S_2386intUP | tgatctaaatccacgaatag | Confirmation of the Δ*bauB* mutant strain |
| A1S_2386intDOWN | gatgaagctattgagtttct |  |
| A1S_2387UPFw | ctgcagtaaagaatatcgggatccgc | Construction of the Δ*bauE* mutant strain |
| A1S_2387UPRv | gatatcccttgatatgaaacatgccg |  |
| A1S_2387DOWNFw | gatatctgaatcatgcaacacctcgg |  |
| A1S_2387DOWNRv | ggatcccaatcatttacccatttgaa |  |
| A1S_2387intUP | tgagtggtatctgcaacttt | Confirmation of the Δ*bauE* mutant strain |
| A1S_2387intDOWN | ttgttctatttctcggtagg |  |
| A1S_2388UPFw | ggggcggccgcatctagaaagtcagcttcat | Construction of the Δ*bauC* mutant strain |
| A1S_2388UPRv | ggggatatcttgccatatttattgctgct |  |
| A1S_2388DOWNFw | ggggatatcttatctgaactccctcaaaa |  |
| A1S_2388DOWNRv | gggctgcaggtatttgctcattattgctc |  |
| A1S_2388intUP | aatgactaaaatcacagtgc | Confirmation of the Δ*bauC* mutant strain |
| A1S_2388intDOWN | tttacaaagtatgcaagggt |  |
| A1S_2389UPFw | gggctgcaggtcagtcagtattggcttat | Construction of the Δ*bauD* mutant strain |
| A1S_2389UPRv | ggggatatcggacttaccgcaggtggt |  |
| A1S_2389DOWNFw | ggggatatcaaatcgcatcagtggcgtg |  |
| A1S_2389DOWNRv | gggggatccgcctgaatgaaatctgactt |  |
| A1S_2389intUP | gccataagagataaatcgct | Confirmation of the Δ*bauD* mutant strain |
| A1S_2389intDOWN | ggaatagggctgtgattttg |  |
| A1S_2390UPFw | ttgcggccgcaaagttaacgacactaaaattc | Construction of the Δ*basB* mutant strain |
| A1S_2390UPRv | cggggtaccccgaaacgtcggttaacttgtt |  |
| A1S_2390DOWNFw | cggggtaccccgaaccttagctcaaatctag |  |
| A1S_2390DOWNRv | cgcggatccgcgaatttggagtatatggcat |  |
| A1S_2390intUP | gacatcaactcattagaaaa | Confirmation of the Δ*basB* mutant strain |
| A1S_2390intDOWN | cttctaaaacacagtcatc |  |
| A1S_2391UPFw | gggctgcaggaacttcccttaagaattcg | Construction of the Δ*basA* mutant strain |
| A1S_2391UPRv | ggggatatcaaaaattgaaatgtgcttaa |  |
| A1S_2391DOWNFw | ggggatatctgctgttcgattggaatttt |  |
| A1S_2391DOWNRv | gggggatccttcaatgaaaatatccgcac |  |
| A1S_2391intUP | acgtgatgaatcgatgtttg | Confirmation of the Δ*basA* mutant strain |
| A1S_2391intDOWN | tagacgatagaattagttcac |  |
| A1S_2392UPFw | gggctgcagccactgccattgaagcaaa | Construction of the Δ*bauF* mutant strain |
| A1S_2392UPRv | ggggatatcatatataacccaagcttaatg |  |
| A1S_2392DOWNFw | ggggatatcgtttcttttacatacaaatagg |  |
| A1S_2392DOWNRv | gggggatccttgcaaaagtgcattcatgg |  |
| A1S_2392intUP | ccacccaacctaatattcaa | Confirmation of the Δ*bauF* mutant strain |
| A1S_2392intDOWN | aaaacttggaaccgaatgg |  |
| A1S_2579UPFw | cattgttgatcttgctgaat | Construction of the Δ*entA* mutant strain |
| A1S_2579UPRv | agaaaattgccttaaccgaa |  |
| A1S_2579DOWNFw | atttctcttgtgtttctgct |  |
| A1S_2579DOWNRv | atttcatattggagcacgaa |  |
| A1S_2579intUP | tttcttgatggtcaactgat | Confirmation of the Δ*entA* mutant strain |
| A1S_2579intDOWN | aggaggtgattaacaatatc |  |
| A1S_2580UPFw | ctcaaaatgatcgactaact | Construction of the Δ*basF/*Δ*fbsC* double mutant strain |
| A1S_2580UPRv | tcaaagtgtgattgcttcat |  |
| A1S_2580DOWNFw | ggataatcttgtttaaacgg |  |
| A1S_2580DOWNRv | ggttcattcaaactaaacag |  |
| A1S_2580intUP | ccatgacaatattgccattt | Confirmation of the Δ*basF/*Δ*fbsC* double mutant strain |
| A1S_2580intDOWN | agttaagcgtttcagaacaa |  |
| A1S_2581UPFw | aaataatctggtggagaagt | Construction of the Δ*basJ/*Δ*fbsB* double mutant strain |
| A1S_2581UPRv | cttgtgaaacttgattaagc |  |
| A1S_2581DOWNFw | aattcataaagcctcctttg |  |
| A1S_2581DOWNRv | gcaggaatttgatttgactt |  |
| A1S_2581intUP | gtagacaccacaaataatca | Confirmation of the Δ*basJ/*Δ*fbsB* double mutant strain |
| A1S_2581intDOWN | gagcttaccgaaatcataaa |  |
| pMo130 site2 Fw | attcatgaccgtgctgac | Confirmation of the plasmid construction |
| pMo130 site2 Rv | cttgtctgtaagcggatg |  |
