# Supplementary material for: In-Depth Analysis of the Role of the Acinetobactin Cluster in the Virulence of Acinetobacter baumannii
Source: Front Microbiol. 2021 Oct 5;12:752070. doi: 10.3389/fmicb.2021.752070 (PMC8524058; doi:10.3389/fmicb.2021.752070)
Supplement: Supplementary file 2 [file Table_2.DOCX]

**Supplementary Table 2.** Genes involved in the biosynthesis and transport of acinetobactin and fimsbactin in *A. baumannii* ATCC 17978.

| **Locus tag** | **Gene name** | **Gene function** |
| --- | --- | --- |
| **ACINETOBACTIN** |  |  |
| A1S_2372 | *basJ* | Biosynthesis: Isochorismate synthetase |
| A1S_2373 | *basI* | Biosynthesis: 4'-phosphopantetheinyl transferase superfamily protein |
| A1S_2374 | *basH* | Biosynthesis: Thioesterase |
| A1S_2375 | *barB* | Efflux: ABC transporter |
| A1S_2376/77/78 | *barA* | Efflux: ABC transporter |
| A1S_2379 | *basG* | Biosynthesis: Histidine decarboxylase |
| A1S_2380 | *basF* | Biosynthesis: 2,3 dihydro-2,3 dihydroxybenzoate synthase |
| A1S_2381 | *basE* | Biosynthesis: 2,3 dihydroxybenzoate- AMP ligase |
| A1S_2382/83 | *basD* | Biosynthesis: Non-ribosomal peptide synthetase |
| A1S_2384 | *basC* | Biosynthesis: putative histamine N-monooxygenase |
| A1S_2385 | *bauA* | Influx: Ferric siderophore receptor protein |
| A1S_2386 | *bauB* | Influx: Ferric acinetobactin transport system periplasmic binding |
| A1S_2387 | *bauE* | Influx: Ferric acinetobactin transport system ATP-binding protein |
| A1S_2388 | *bauC* | Influx: Ferric acinetobactin transport system permease |
| A1S_2389 | *bauD* | Influx: Ferric acinetobactin transport system permease |
| A1S_2390 | *basB* | Biosynthesis: Non-ribosomal peptide synthetase |
| A1S_2391 | *basA* | Biosynthesis: Non-ribosomal peptide synthetase |
| A1S_2392 | *bauF* | Transport: Siderophore-interacting protein |
| A1S_2579 | *entA/fbsD* | Biosynthesis: 2,3-dihydro-2,3-dihydroxybenzoate dehydrogenase |
| **FIMSBACTIN** |  |  |
| A1S_2562 | *fbsQ* | Efflux: MATE family efflux transporter |
| A1S_2563/64 | *fbsP* | Influx: Siderophore-interacting protein |
| A1S_2565 | *fbsO* | Efflux: MFS transporter |
| A1S_2566 | *fbsN* | Influx: TonB-dependent siderophore receptor |
| A1S_2567 | *fbsM* | Biosynthesis: Thioesterase |
| A1S_2568 | *fbsL* | Biosynthesis: 4'-phosphopantetheinyl transferase superfamily protein |
| A1S_2569 | HP | Hypothetical protein |
| A1S_2570 | *fbsK* | Biosynthesis: Acetyltransferase |
| A1S_2571 | *fbsJ* | Biosynthesis: Ornithine decarboxylase |
| A1S_2572 | *fbsI* | Biosynthesis: SidA/IucD/PvdA family monooxygenase |
| A1S_2573/74 | *fbsH* | Biosynthesis: 2,3-dihydroxybenzoate-AMP ligase |
| A1S_2575 | *fbsG* | Biosynthesis: Non-ribosomal peptide synthetase |
| A1S_2576/77 | *fbsF* | Biosynthesis: Non-ribosomal peptide synthetase |
| A1S_2578 | *fbsE* | Biosynthesis: Non-ribosomal peptide synthetase |
| A1S_2579 | *fbsD/entA* | Biosynthesis: 2,3-dihydro-2,3-dihydroxybenzoate dehydrogenase |
| A1S_2580 | *fbsC* | Biosynthesis: 2,3 dihydro-2,3 dihydroxybenzoate synthase |
| A1S_2581 | *fbsB* | Biosynthesis: Isochorismate synthetase |
| A1S_2582 | *fbsA* | Regulation: AraC family transcriptional regulator |
