# Supplementary material for: In-Depth Analysis of the Role of the Acinetobactin Cluster in the Virulence of Acinetobacter baumannii
Source: Front Microbiol. 2021 Oct 5;12:752070. doi: 10.3389/fmicb.2021.752070 (PMC8524058; doi:10.3389/fmicb.2021.752070)
Supplement: Supplementary file 3 [file Table_3.DOCX]

**Supplementary Table 3.** Comparation between potential redundant biosynthetic genes of the acinetobactin and fimsbactin clusters in *A. baumannii* ATCC 17978.

| **ACINETOBACTIN** | | | **FIMSBACTIN** | | | **Gen function** | **Final function** |
| --- | --- | --- | --- | --- | --- | --- | --- |
| **Locus tag** | **Gene name** | **Protein domains** | **Locus tag** | **Gene name** | **Protein domains** |  |  |
| A1S_2372 | *basJ* | Isochr_syn (TIGR00543) | A1S_2581 | *fbsB* | Isochr_syn (TIGR00543) | Isochorismate synthetase | DHBA biosynthesis |
| A1S_2373 | *basI* | EntD (COG2977), 4PPT_N (pfam17837) | A1S_2568 | *fbsL* | EntD (COG2977), 4PPT_N (pfam17837) | 4'-phosphopantetheinyl transferase superfamily protein | NRPS assembly system |
| A1S_2374 | *basH* | Thioesterase (pfam00975) | A1S_2567 | *fbsM* | Thioesterase (pfam00975) | Thioesterase | NRPS assembly system |
| A1S_2379 | *basG* | tyr_de_CO2_Arch (TIGR03812) |  |  |  | Histidine decarboxylase | N-hydroxyhistamine biosynthesis |
| A1S_2380 | *basF* | Isochorismatase (cd01013), EntB (COG1535) | A1S_2580 | *fbsC* | Isochorismatase (cd01013), EntB (COG1535) | 2,3 dihydro-2,3 dihydroxybenzoate synthase | DHBA biosynthesis |
| A1S_2381 | *basE* | EntE (PRK10946), DHB_AMP_lig (TIGR02275) | A1S_2574 | *fbsH* | EntE (PRK10946), DHB_AMP_lig (TIGR02275) | 2,3 dihydroxybenzoate- AMP ligase | DHBA activation |
| A1S_2382/83 | *basD* | Cyc_NRPS (cd19535), EntF (COG1020) | A1S_2577/78 | *fbsE* | Cyc_NRPS (cd19535), EntF (COG1020) | Non-ribosomal peptide synthetase | NRPS assembly system |
| A1S_2384 | *basC* | Histamin_N_OH (TIGR04439) |  |  |  | Putative histamine N-monooxygenase | N-hydroxyhistamine biosynthesis |
| A1S_2390 | *basB* | LCL_NRPS-like (cd19540) | A1S_2575 | *fbsG* | LCL_NRPS-like (cd19540) | Non-ribosomal peptide synthetase | NRPS assembly system |
| A1S_2391 | *basA* | A_NRPS_TlmIV_like (cd12114), EntF (COG1020) | A1S_2576 | *fbsF* | A_NRPS_TlmIV_like (cd12114), EntF (COG1020) | Non-ribosomal peptide synthetase | NRPS assembly system |
