# Supplementary material for: In-Depth Analysis of the Role of the Acinetobactin Cluster in the Virulence of Acinetobacter baumannii
Source: Front Microbiol. 2021 Oct 5;12:752070. doi: 10.3389/fmicb.2021.752070 (PMC8524058; doi:10.3389/fmicb.2021.752070)
Supplement: Supplementary file 4 [file Image_1.pdf]

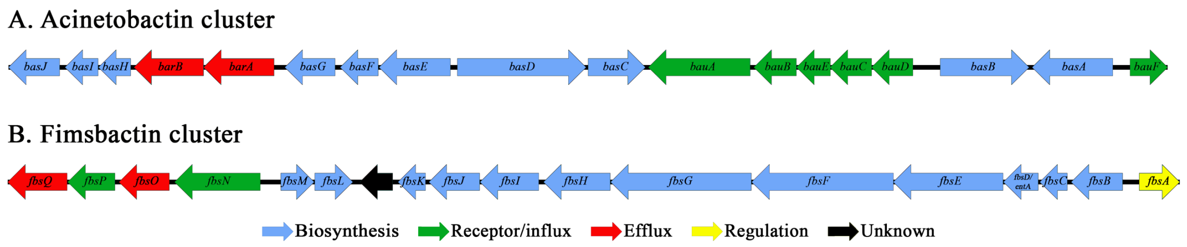

Supplementary Figure 1. Genetic organization of the **(A)** acinetobactin and **(B)** fimsbactin clusters of *A. baumannii* ATCC 17978.
