# Supplementary material for: In-Depth Analysis of the Role of the Acinetobactin Cluster in the Virulence of Acinetobacter baumannii
Source: Front Microbiol. 2021 Oct 5;12:752070. doi: 10.3389/fmicb.2021.752070 (PMC8524058; doi:10.3389/fmicb.2021.752070)
Supplement: Supplementary file 5 [file Image_2.PDF]

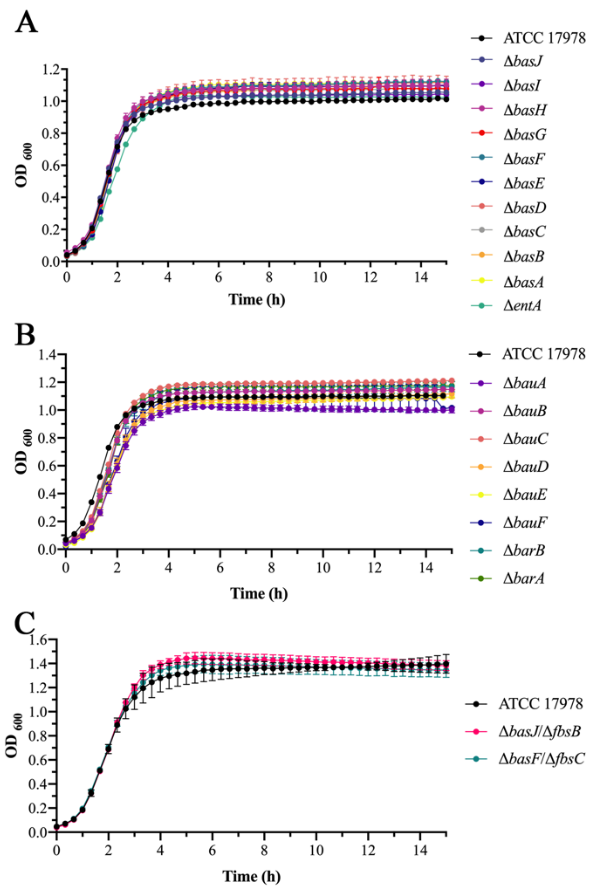

Supplementary Figure 2. Growth curves performed in MH under normal conditions. **(A)** Growth curves of *A. baumannii* ATCC 17978 and its isogenic mutant derivative strains lacking the genes involved in the acinetobactin biosynthesis. **(B)** Growth curves of *A. baumannii* ATCC 17978 and its isogenic mutant derivative strains lacking the genes involved in the acinetobactin transport. **(C)** Growth curves of *A. baumannii* ATCC 17978 and the Δ*basJ*/Δ*fbsB* and Δ*basF*/Δ*fbsC* double mutant strains. Three independent biological replicates were performed.
