# Supplementary material for: In-Depth Analysis of the Role of the Acinetobactin Cluster in the Virulence of Acinetobacter baumannii
Source: Front Microbiol. 2021 Oct 5;12:752070. doi: 10.3389/fmicb.2021.752070 (PMC8524058; doi:10.3389/fmicb.2021.752070)
Supplement: Supplementary file 6 [file Image_3.PDF]

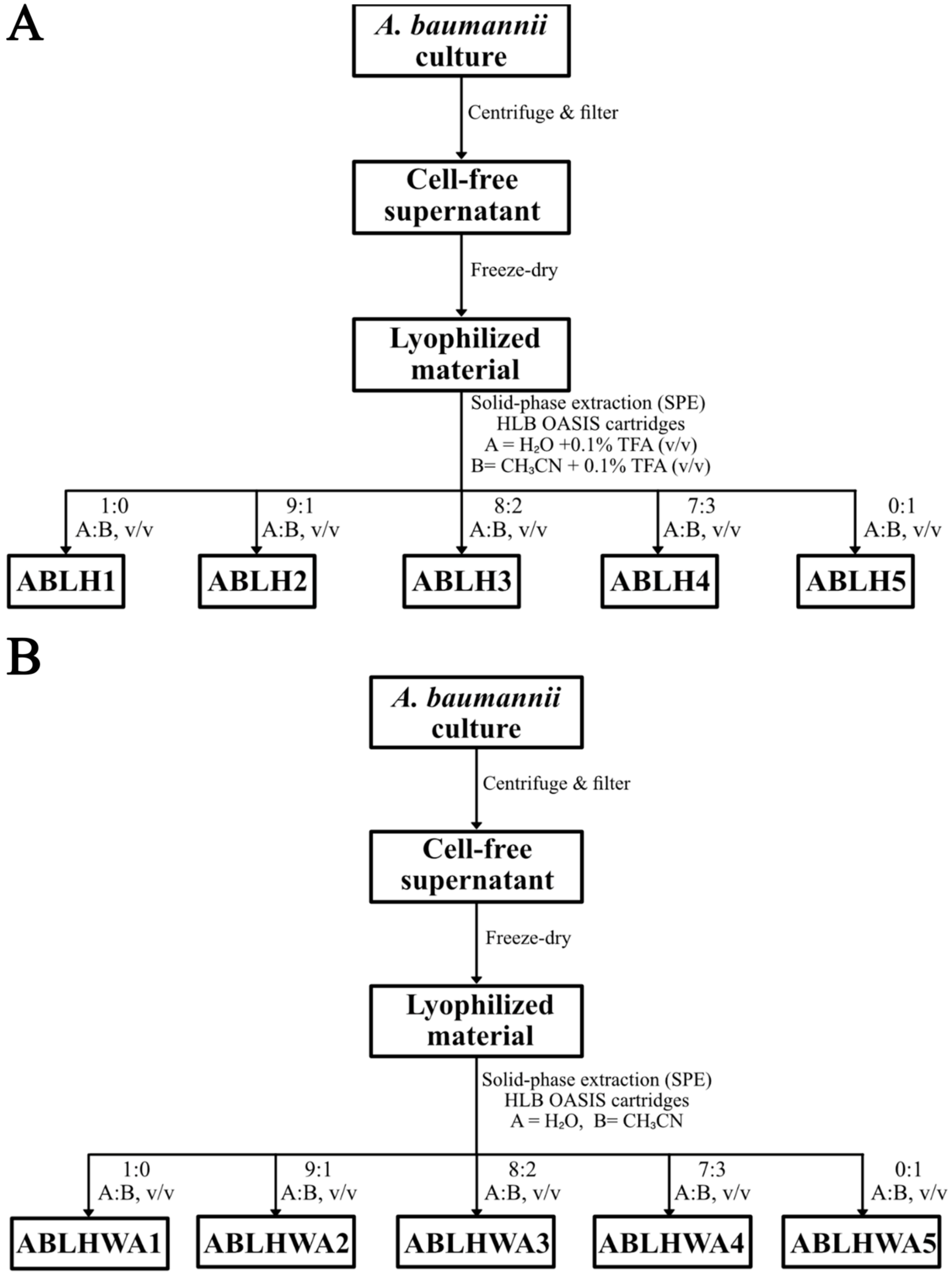

Supplementary Figure 3. Fractionation flowchart of *A. baumannii* wild-type and mutant strains cultures under **(A)** acidic conditions and **(B)** non-acidic conditions.
