# Supplementary material for: In-Depth Analysis of the Role of the Acinetobactin Cluster in the Virulence of Acinetobacter baumannii
Source: Front Microbiol. 2021 Oct 5;12:752070. doi: 10.3389/fmicb.2021.752070 (PMC8524058; doi:10.3389/fmicb.2021.752070)
Supplement: Supplementary file 7 [file Image_4.PDF]

A

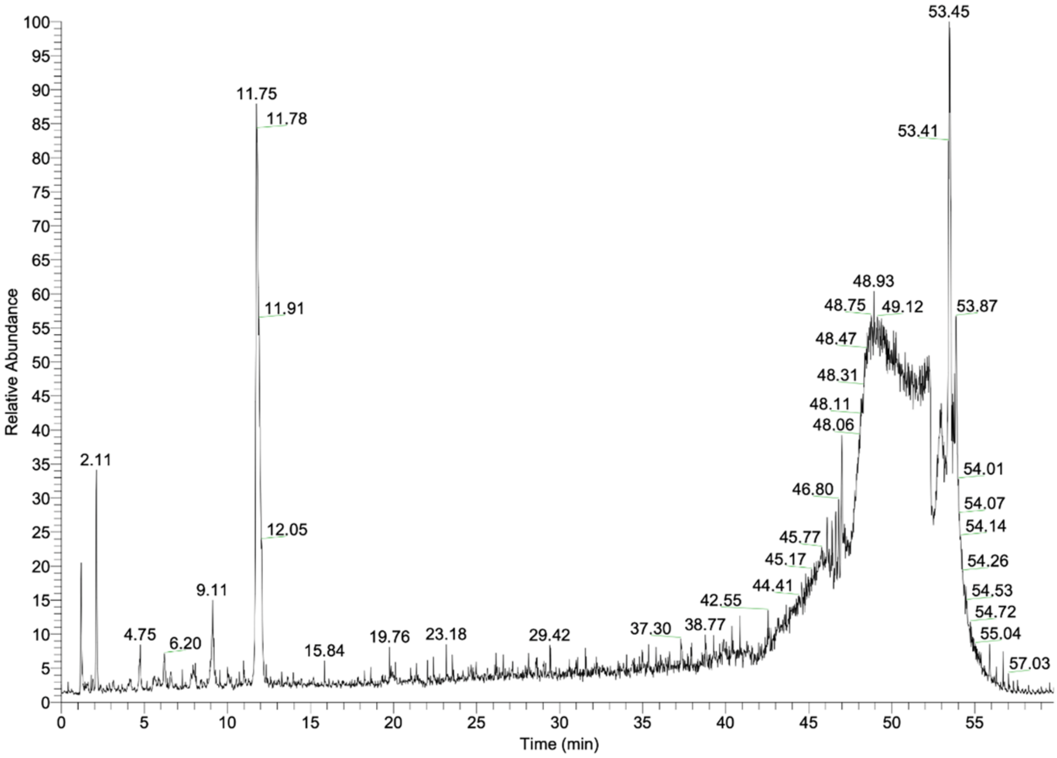

**Column**	Discovery HS-F5
**Mobile Phase**	A (0.1% TFA in deionized water) and B (0.1% TFA in CH_3CN)
**Gradient**	1) 40 minutes from 10 to 50% of B, 2) 5 minutes from 50 to 100 % of B, 3) isocratic step of 5 minutes at 100% of B, 4) 5 minutes from 100 to 10 % of B, 5) isocratic step of 5 minutes at 10% of B.
**Flow rate**	1 mL/min
**UV detector**	254, 280 and 313 nm
**MS detector**	Full positive ion mode

B

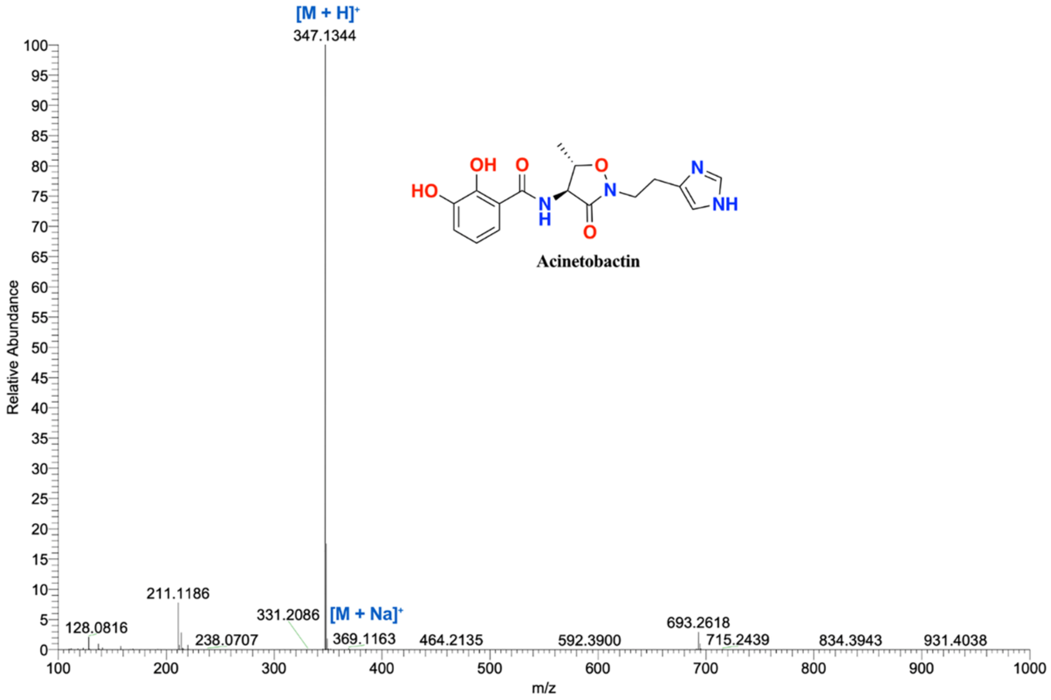

Supplementary Figure 4. (A) Total ion current (TIC) chromatogram of the ABLH3 fraction eluted with 8:2 of $H_2O:CH_3CN$ (v/v), each containing 0.1% TFA, from the *A. baumannii* wild-type cell-free supernatant and HPLC/HRMS conditions used for the analysis. **(B)** (+)-HR-ESIMS of chromatographic peak eluted with the rt = 12 minutes of the ABHL3 fraction from the *A. baumannii* wild-type cell-free supernatant and identified as acinetobactin.
