# Supplementary material for: In-Depth Analysis of the Role of the Acinetobactin Cluster in the Virulence of Acinetobacter baumannii
Source: Front Microbiol. 2021 Oct 5;12:752070. doi: 10.3389/fmicb.2021.752070 (PMC8524058; doi:10.3389/fmicb.2021.752070)
Supplement: Supplementary file 8 [file Image_5.PDF]

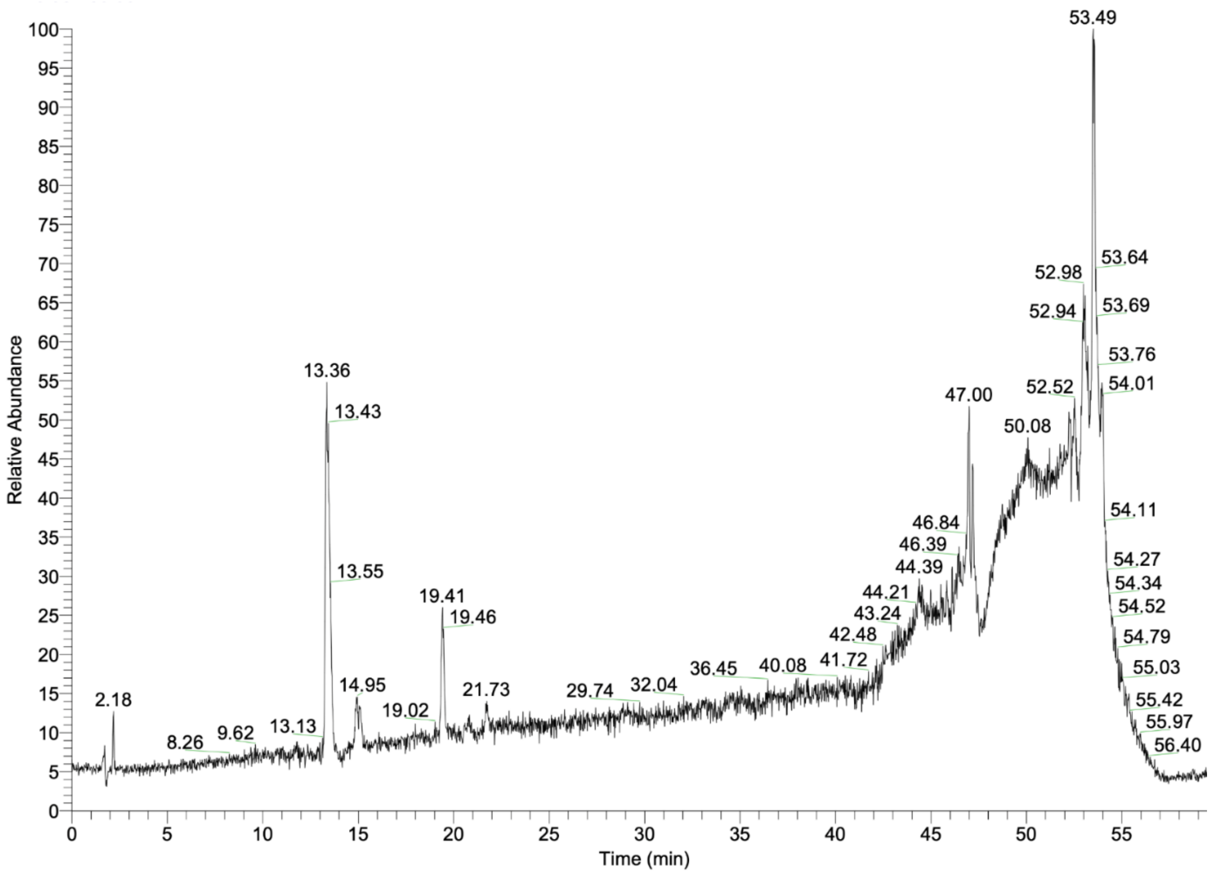


**Supplementary Figure 5.** Total ion current (TIC) chromatogram of the ABLH5 fraction eluted with 0:1 of $H_2O$:$CH_3CN$ (v/v), each containing 0.1% TFA, from the *A. baumannii* wild-type cell-free supernantant. HPLC/HRMS conditions used for the analysis were the same described in Supplementary Figure S4.
