# Supplementary material for: In-Depth Analysis of the Role of the Acinetobactin Cluster in the Virulence of Acinetobacter baumannii
Source: Front Microbiol. 2021 Oct 5;12:752070. doi: 10.3389/fmicb.2021.752070 (PMC8524058; doi:10.3389/fmicb.2021.752070)
Supplement: Supplementary file 9 [file Image_6.PDF]

A

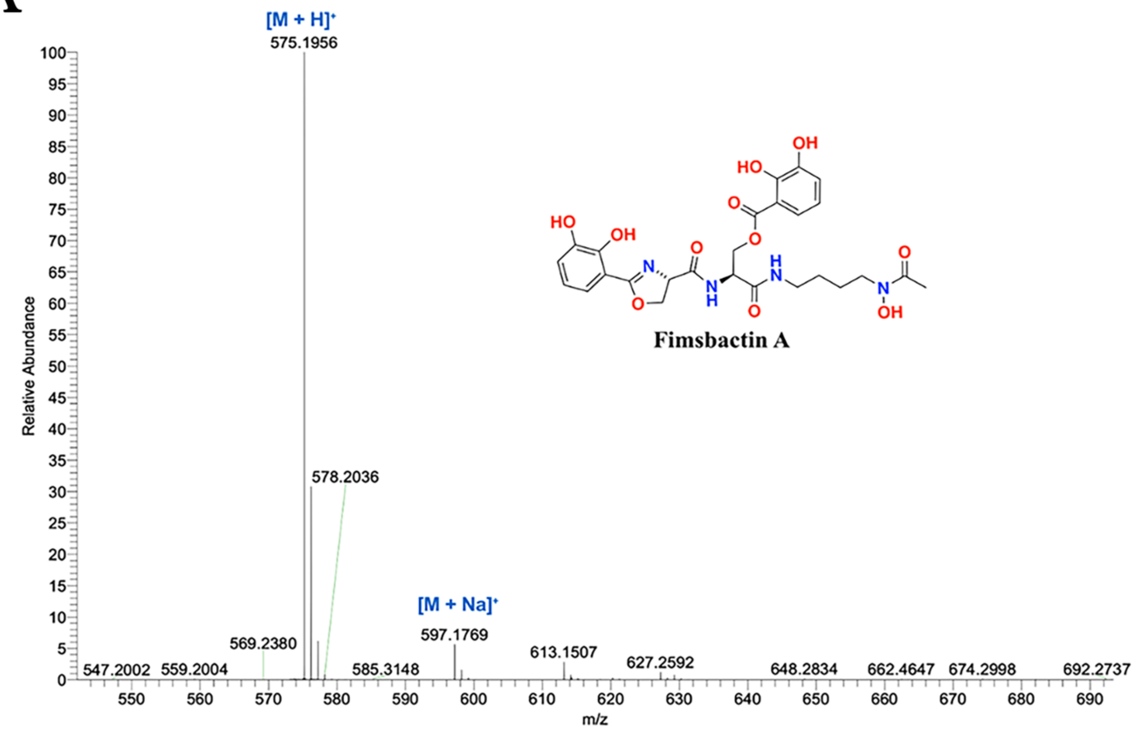

B

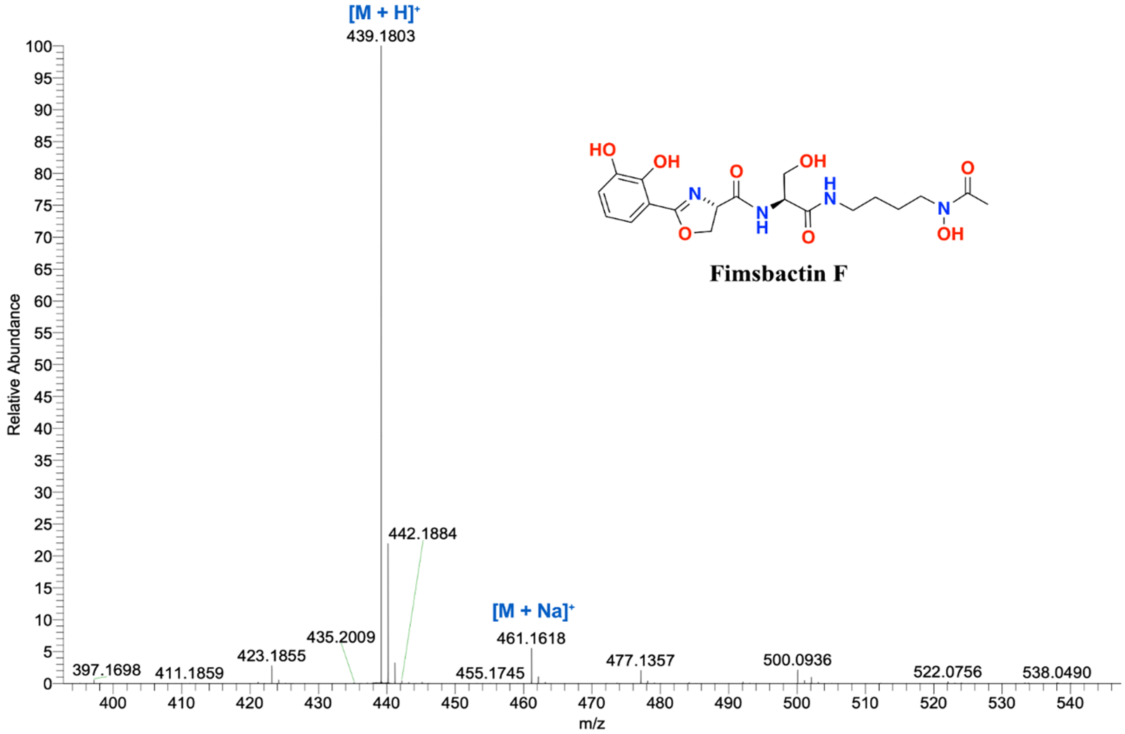

Supplementary Figure 6. (+)-HR-ESIMS of **(A)** fimsbactin A and **(B)** fimsbactin F detected in the chromatographic peak eluted at the retention time 19.4 minutes of ABLH5 fraction from the *A. baumannii* wild-type cell-free supernatant.
