# Supplementary material for: In-Depth Analysis of the Role of the Acinetobactin Cluster in the Virulence of Acinetobacter baumannii
Source: Front Microbiol. 2021 Oct 5;12:752070. doi: 10.3389/fmicb.2021.752070 (PMC8524058; doi:10.3389/fmicb.2021.752070)
Supplement: Supplementary file 10 [file Image_7.PDF]

A

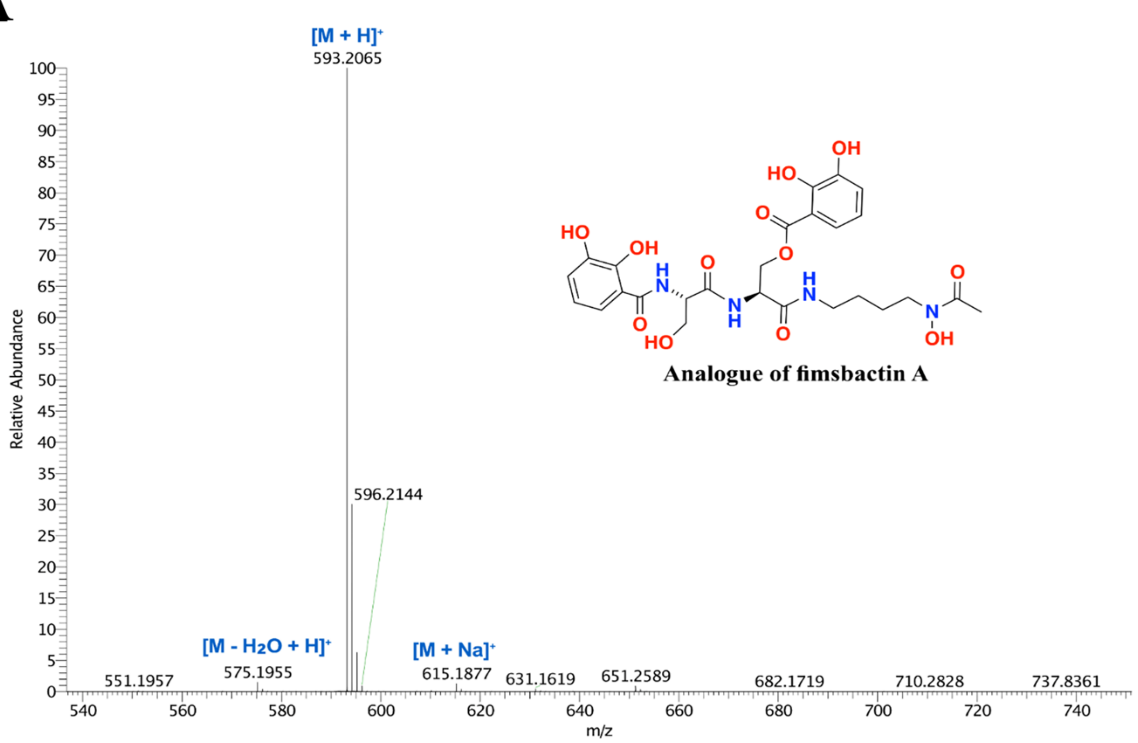

B

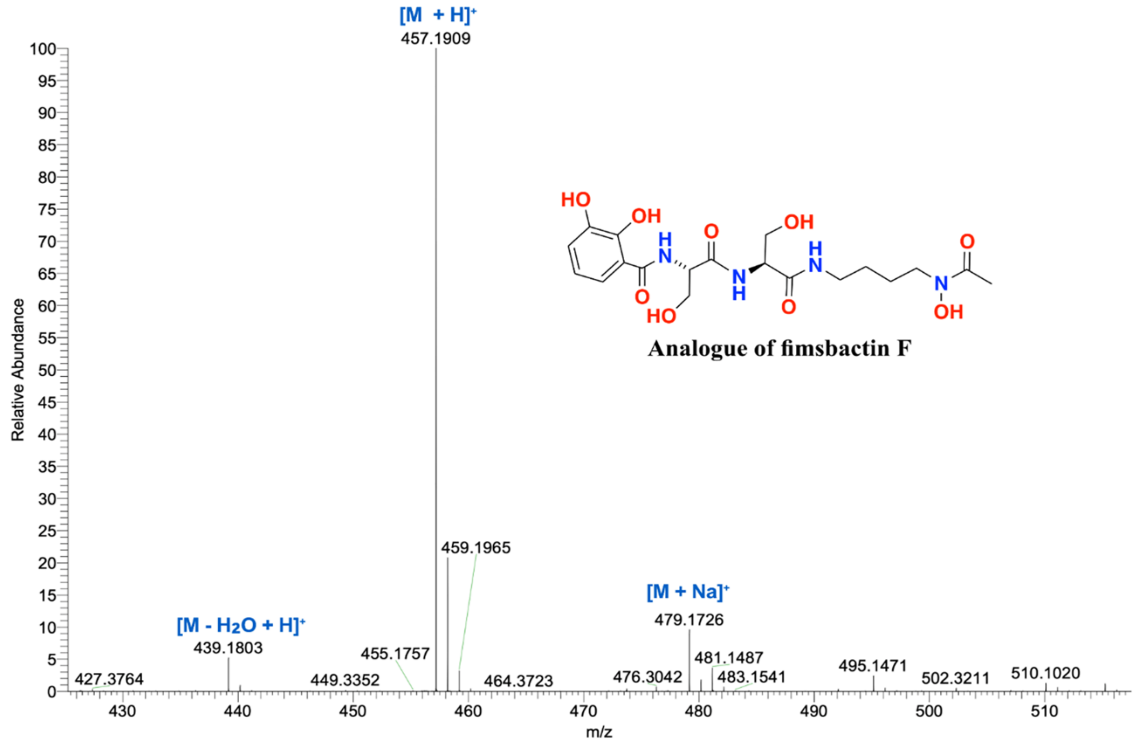

Supplementary Figure 7. (+)-HR-ESIMS of the **(A)** fimsbactin A-derived analogue and **(B)** fimsbactin F-derived analogue detected in the chromatographic peak eluted at the retention time 13.4 minutes of ABLH5 fraction from the *A. baumannii* wild-type cell-free supernatant.
