# Supplementary material for: In-Depth Analysis of the Role of the Acinetobactin Cluster in the Virulence of Acinetobacter baumannii
Source: Front Microbiol. 2021 Oct 5;12:752070. doi: 10.3389/fmicb.2021.752070 (PMC8524058; doi:10.3389/fmicb.2021.752070)
Supplement: Supplementary file 11 [file Image_8.PDF]

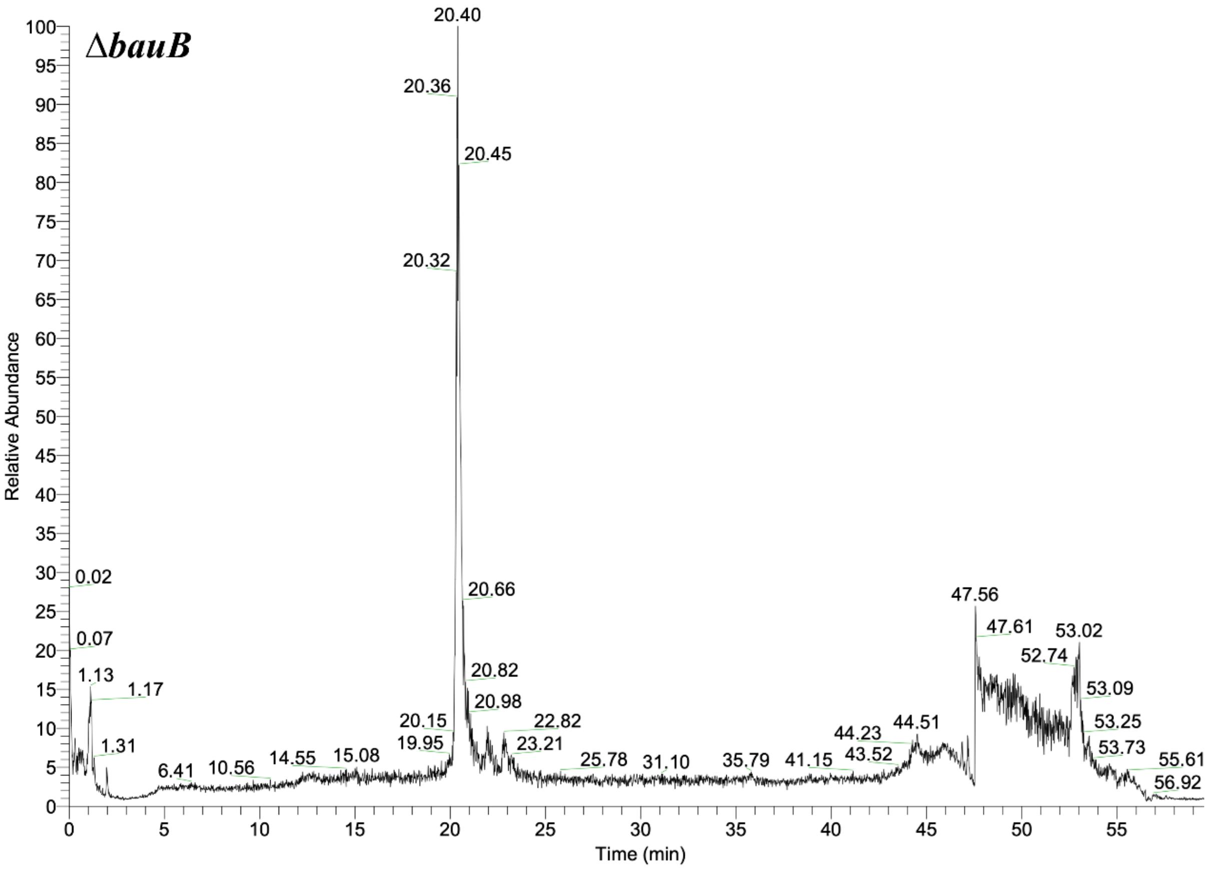

**Column**	Discovery HS-F5
**Mobile Phase**	A (Deionized water) and B (CH_3CN)
**Gradient**	1) 40 minutes from 10 to 50% of B, 2) 5 minutes from 50 to 100 % of B, 3) isocratic step of 5 minutes at 100% of B, 4) 5 minutes from 100 to 10 % of B, 5) isocratic step of 5 minutes at 10% of B.
**Flow rate**	1 mL/min
**UV detector**	254, 280 and 313 nm
**MS detector**	Full positive ion mode

Supplementary Figure 8. Total ion current (TIC) chromatogram of ABLHWA5 fraction eluted with 0:1 of H_2O:CH_3CN (v/v) from the *A. baumannii* mutant strain Δ*bauB* and HPLC/HRMS conditions used for the analysis
