# Supplementary material for: In-Depth Analysis of the Role of the Acinetobactin Cluster in the Virulence of Acinetobacter baumannii
Source: Front Microbiol. 2021 Oct 5;12:752070. doi: 10.3389/fmicb.2021.752070 (PMC8524058; doi:10.3389/fmicb.2021.752070)
Supplement: Supplementary file 16 [file Image_13.PDF]

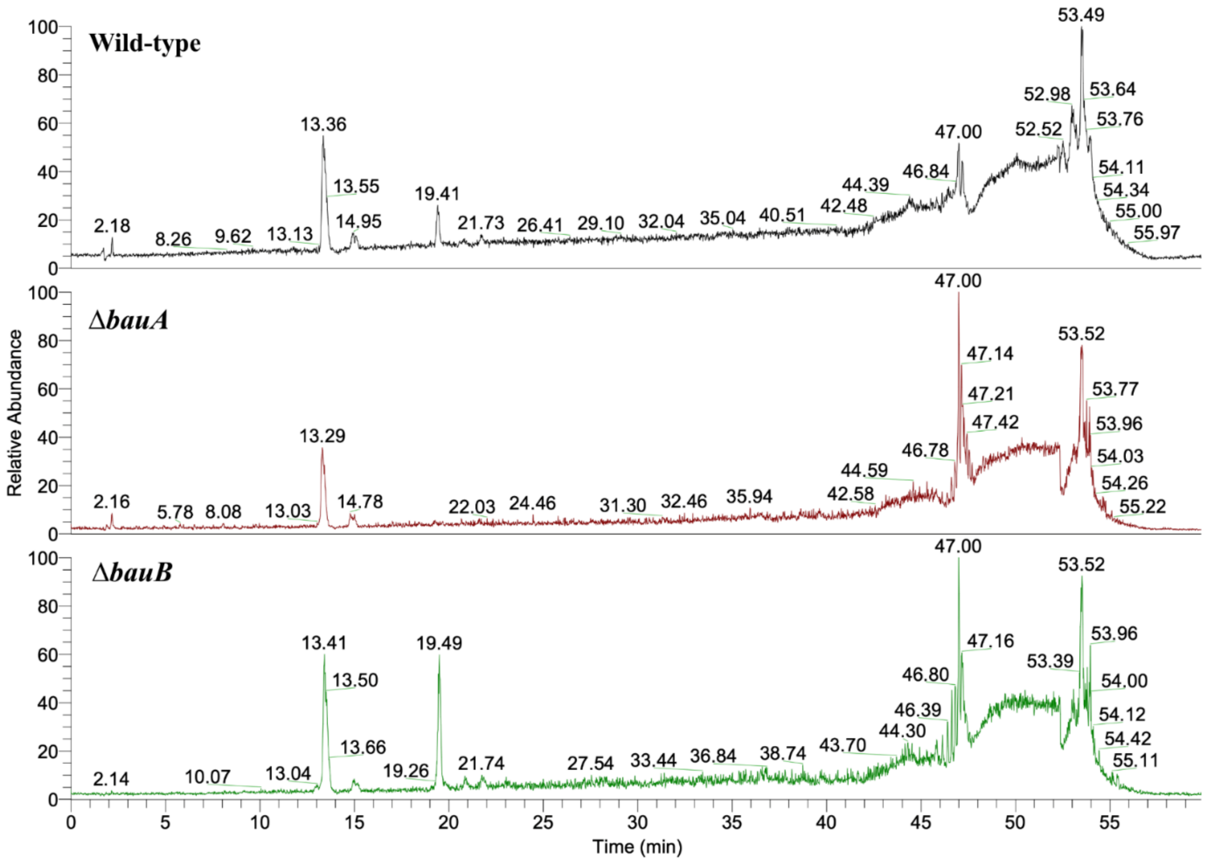

Supplementary Figure 13. Comparison between the total ion current (TIC) chromatographic profiles of the ABHL5 fractions of *A.baumanii* wild-type and mutant strains lacking genes involved in the influx of acinetobactin.
